# Whole blood microRNA levels associate with glycemic status and correlate with target mRNAs in pathways important to type 2 diabetes

**DOI:** 10.1038/s41598-019-43793-4

**Published:** 2019-06-20

**Authors:** Nina Mononen, Leo-Pekka Lyytikäinen, Ilkka Seppälä, Pashupati P. Mishra, Markus Juonala, Melanie Waldenberger, Norman Klopp, Thomas Illig, Jaana Leiviskä, Britt-Marie Loo, Reijo Laaksonen, Niku Oksala, Mika Kähönen, Nina Hutri-Kähönen, Olli Raitakari, Terho Lehtimäki, Emma Raitoharju

**Affiliations:** 1 Department of Clinical Chemistry, Pirkanmaa Hospital District, Fimlab Laboratories, and the Finnish Cardiovascular Research Center, Tampere, Faculty of Medicine and Health Technology, Tampere University, Tampere, Finland; 2 Division of Medicine, Turku University Hospital, and Department of Medicine, University of Turku, Turku, Finland; 3 Research Unit of Molecular Epidemiology, Helmholtz Zentrum, German Research Center for Environmental Health, Munich, Germany; 4 Hannover Unified Biobank, Hannover Medical School, Hannover, Germany; 5 Institute for Human Genetics, Hannover Medical School, Hanover, Germany; 6 Department of Clinical Chemistry, University of Helsinki and Helsinki University Hospital HUSLAB, Helsinki, Finland; 7 Joint Clinical Biochemistry Laboratory of the University of Turku and Turku University Central Hospital and Department of Chronic Disease Prevention, National Institute for Health and Welfare, Turku, Finland; 8 Centre for Vascular Surgery and Interventional Radiology, Tampere University Hospital, Tampere, Finland; 9 Department of Clinical Physiology, Tampere University Hospital, and Faculty of Medicine and Health Technology, Tampere University, Tampere, Finland; 10 Department of Pediatrics, Tampere University and Tampere University Hospital, Tampere, Finland; 11 Research Centre for Applied and Preventive Cardiovascular Medicine, University of Turku, Turku, Finland; 12 Department of Clinical Physiology and Nuclear Medicine and Centre for Population Health Research, University of Turku and Turku University Hospital, Turku, Finland

**Keywords:** miRNAs, Pre-diabetes

## Abstract

We analyzed the associations between whole blood microRNA profiles and the indices of glucose metabolism and impaired fasting glucose and examined whether the discovered microRNAs correlate with the expression of their mRNA targets. MicroRNA and gene expression profiling were performed for the Young Finns Study participants (n = 871). Glucose, insulin, and glycated hemoglobin (HbA1c) levels were measured, the insulin resistance index (HOMA2-IR) was calculated, and the glycemic status (normoglycemic [n = 534]/impaired fasting glucose [IFG] [n = 252]/type 2 diabetes [T2D] [n = 24]) determined. Levels of hsa-miR-144-5p, -122-5p, -148a-3p, -589-5p, and hsa-let-7a-5p associated with glycemic status. hsa-miR-144-5p and -148a-3p associated with glucose levels, while hsa-miR-144-5p, -122-5p, -184, and -339-3p associated with insulin levels and HOMA2-IR, and hsa-miR-148a-3p, -15b-3p, -93-3p, -146b-5p, -221-3p, -18a-3p, -642a-5p, and -181-2-3p associated with HbA1c levels. The targets of hsa-miR-146b-5p that correlated with its levels were enriched in inflammatory pathways, and the targets of hsa-miR-221-3p were enriched in insulin signaling and T2D pathways. These pathways showed indications of co-regulation by HbA1c-associated miRNAs. There were significant differences in the microRNA profiles associated with glucose, insulin, or HOMA-IR compared to those associated with HbA1c. The HbA1c-associated miRNAs also correlated with the expression of target mRNAs in pathways important to the development of T2D.

## Introduction

Globally, approximately 422 million adults were living with diabetes in 2014, and the age-standardized prevalence has more than doubled since 1980^1^. The majority of the cases of diabetes in adults are of type 2 (T2D), and in addition to increasing rates in adults, T2D accounts for 8–45% of all new cases of diabetes reported among children and adolescents^2^. The development of T2D is asymptomatic, and disease that already fulfils the diagnostic criteria of T2D often remains subclinical for a long period. Prior to T2D, individuals can reside in the high-risk state of prediabetes, defined as impaired fasting glucose (IFG) or impaired glucose tolerance^3^.

T2D is a complex multi-organ disease with many interrelated dysfunctions. Insulin resistance in the skeletal muscles and in adipose tissue is followed initially by an increase in insulin production in pancreatic β-cells. The failure to respond to the increased insulin demand will eventually lead to progressive β-cell failure, hyperglycemia, and glucose toxicity. The disease is also characterized by the accumulation of fat in the liver, as adipose tissue lipolysis is not sufficiently inhibited by insulin in type 2 diabetes. The pathogenesis of T2D has been shown to be heterogenic, and the progression of the disease, for example, differs according to the age of onset^4^. The risk of complications also varies according to ethnicity^5^ as well as the level of control of the glycemic status^6^.

The rise in the prevalence of T2D is tied to the increasing rates of obesity. Positive T2D family history also increases the risk for developing the disease^7^, even though independent variations in the DNA code have been shown to explain only a small proportion of the heritability of T2D^8,9^. Recently, it has been hypothesized that the effect could also be conveyed by epigenetic changes and that these changes could be mediated by predisposing individuals to the risk of insulin resistance during gestation, and possibly even by the inter- and transgenerational transmission of the disorder^7^.

MicroRNAs (miRNAs, miRs) are small non-protein coding RNAs. They are known to regulate gene expression at the post-transcriptional level by binding to target mRNAs and affecting their translation. MicroRNAs can control several genes, and individual mRNAs can be bound by several miRNAs—miRNAs can thus establish wide regulatory networks affecting several metabolic processes. Research on T2D and miRNAs in humans has focused on the identification of possible biomarkers for established T2D. The results have been inconsistent, mainly due to the small populations studied, different starting materials and profiling methods, preselected pools of miRNAs, and the heterogeneity of T2D as a disease^10^. A meta-analysis by Zhu *et al*. indicated that miR-29a, -34a, -375, -103, -107, -132, -142-3p, and -144 could be potential circulatory biomarkers (plasma, serum, peripheral blood mononuclear cells, and whole blood as profiling samples) for T2D^10^, while another meta-analysis reported that levels of miR-320a, -142-3p, -222, -29a, -27a, and -375 increased and levels of miR-197, -20b, -17, and -652 decreased in individuals with T2D^11^.

In contrast to these studies, more long-term solutions for the T2D epidemic could be reached by identifying individuals early in the prediabetic state and by understanding the function of miRNAs in both the normoglycemic (NG) and prediabetic state. However, miRNAs in prediabetes have been studied far less. Villard *et al*. showed that only circulatory miR-29a, -192, and -126 were consistently dysregulated in individuals with prediabetes^11^. Although we have seen interesting results—indicating, for example, changes in serum miR-192, -193b^12^ and -126 in response to glycemic status and intervention—most studies in humans on miRNAs and prediabetes have been small case/control studies (n < 100)^12–17^ or/and no multiple testing correction has been utilized^12–14,18^, and/or they were performed with only preselected miRNAs^14,17–19^.

The aims of this study were to (**i**) analyze the difference in the whole blood miRNA expression in individuals with and without IFG in the large (n = 871; individuals with IFG = 252 and those with T2D = 24) population-based Young Finns Study (YFS) cohort (aged 34–49 years) with individuals currently approaching the age when T2D is most frequently diagnosed; (ii) to study the association of glucose, insulin, and glycated hemoglobin (HbA1c) levels with the HOMA2 insulin resistance (HOMA-IR) index and whole blood miRNA levels; (iii) to look for insight into the cellular origins of the miRNAs of interest by correlating their levels with the blood cell counts; (iv) to investigate whether these miRNAs of interest correlate with their predicted targets and whether the correlated targets are enriched in specific biological pathways; and (v) to see whether the miRNAs of interest together may co-regulate these pathways.

## Results

### The levels of five miRNAs are associated with glycemic status

**Figure 1:**
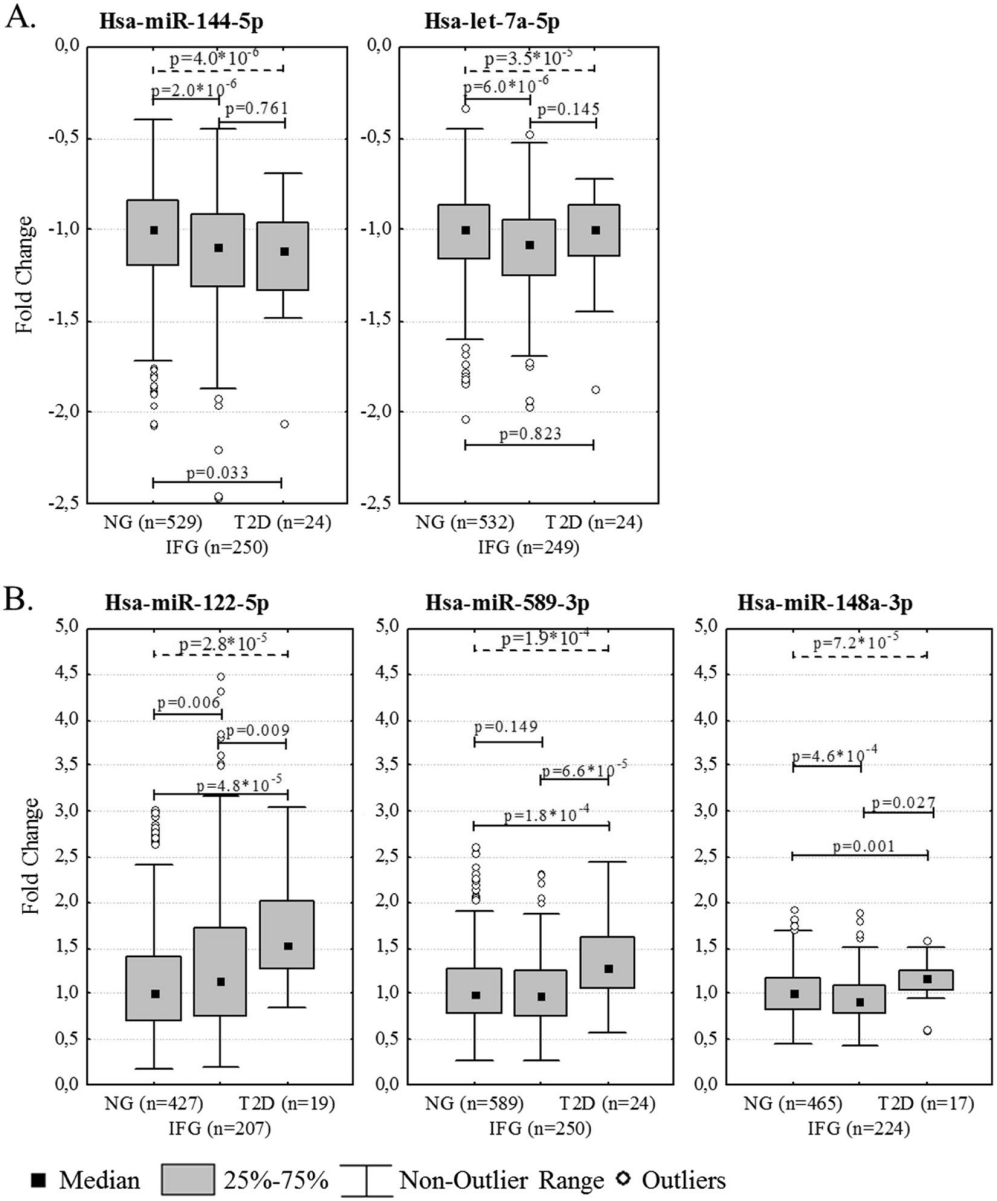
Blood levels of hsa-miR-144- 5p, -let-7a-5p (**A**), -122-5p, -589-3p, and -148a-3p (**B**) in normoglycemic individuals (NG), individuals with impaired fasting glucose (IFG) and individuals with type 2 diabetes (T2D). The trend over groups is analyzed using the Kruskal-Wallis test (dash line) and the differences between groups by the Mann-Whitney U test (solid line). Hsa-miR-144-5p and Let-7a-5p are significantly (Bonferroni corrected p < 0.05) down-regulated in IFG vs. NG (**A**), while hsa-miR-122-5p and 589-3p were significantly up-regulated in T2D vs. NG (**B**).

The Kruskal-Wallis test on glycemic status groups (NG, IFG, and T2D) showed a significant association (p_c_ < 0.05) for the levels of hsa-miR-144-5p, -122-5p, -148a-3p, -589-5p, and hsa-let7a-5p. Hsa-miR-144-5p and hsa-let7a-5p were significantly down-regulated in individuals with IFG (vs. NG) (p_c_ < 0.05, FC = 0.91 and FC = 0.93, respectively) (Fig. 1A, Table 1, Supplementary Table S1). Both of the miRNAs were significant (p < 0.05) statistical predictors of IFG (vs. NG) also in the fully adjusted model 2. Hsa-miR-148a-3p was also down-regulated in individuals with IFG (FC = 0.92) and up-regulated in those with T2D (vs. NG) (FC = 1.12) (Fig. 1A), but the significance did not survive multiple testing correction and this miRNA was thus not included in further regression analysis.

**Table 1:** Significant (p_c_ < 0.05) associations between miRNAs and individuals’ glycemic status (normoglycemic [NG/impaired fasting glucose [IFG]/type 2 diabetes [T2D]).

	IFG vs. NG		T2D vs. NG		T2D vs. IFG	
	p-value	FC/OR	p-value	FC/OR	p-value	FC/OR
**hsa-miR-144-5p**						
*U-test*	**2**.35*10^−**6**^	**0**.**91**	0.033	0.90		
*Model 1*	**7**.42*10^−**5**^	**0**.**71**	0.043	0.63		
*Model 2*	3.64*10^−4^	0.73				
**hsa-let-7a-5p**						
*U-test*	**6**.39*10^−**6**^	**0**.**93**				
*Model 1*	3.00*10^−4^	0.74				
*Model 2*	4.50*10^−4^	0.73				
**hsa-miR-122-5p**						
*U-test*	0.006	1.14	**4**.80*10^−**5**^	**1**.**53**	0.009	1.34
*Model 1*			0.002	2.68	0.042	1.64
*Model 2*			0.010	2.48		
**hsa-miR-589-3p**						
*U-test*			**1**.82*10^−**4**^	**1**.**29**	**6**.60*10^−**5**^	**1**.**32**
*Model 1*			2.34*10^−4^	2.53	2.27*10^−4^	2.51
*Model 2*			3.70*10^−4^	2.80	**1**.65*10^−**4**^	**2**.**83**

Associations are evaluated with the Mann-Whitney U test and stepwise logistic regression models. Only p-values smaller than 0.05 are shown here and those with a p_c_ < 0.05 are indicated by bold font. *Multiply sign. Fold changes (FC) describe the magnitude of the difference with the Mann-Whitney U test, while odds ratios (OR) were calculated with regression models. All p-values, numbers of samples, and 95% CIs are shown in Supplementary Table 1.

Statistical model: U-test = Mann-Whitney U test; Model 1 = stepwise logistic regression model including miRNA of interest (one by one), age, sex, and BMI; Model 2 = Model 1 + leukocyte, erythrocyte, and thrombocyte count, in addition to total cholesterol, LDL, HDL, and triglyceride levels, as well as alcohol consumption, and history of smoking or hypertension.

When comparing T2D individuals with NG individuals, hsa-miR-122-5p and hsa-miR-589-3p were up-regulated (p_c_ < 0.05, FC = 1.53 and FC = 1.29, respectively) (Fig. 1B, Table 1, Supplementary Table S1). Both of these miRNAs were also up-regulated (p < 0.05 but p_c_ > 0.05) in T2D, in comparison to individuals with IFG (FC = 1.34 and FC = 1.32). Hsa-miR-589-3p was an independent statistical predictor of T2D when compared to NG or IFG individuals in fully adjusted model 2, and hsa-miR-122-5p was an independent statistical predictor of T2D when compared to NG individuals also in the fully adjusted model, but not when compared to individuals with IFG (Fig. 1A, Table 1, Supplementary Table S1).

### Twelve miRNAs significantly associate with glucose and HbA1c levels and/or indicators of insulin resistance

In addition to being down-regulated in individuals with prediabetes, hsa-miR-144-5p also correlated inversely (p_c_ < 0.05) with insulin levels and the HOMA2 index. This miRNA also associated with serum glucose levels in the fully adjusted model 2 (p < 0.05), but adding triglycerides to the model predicting insulin and HOMA2 index levels abolished the association. Further analysis showed that this miRNA correlated even more significantly with serum triglyceride levels (p = 7.3*10^−20^, r = −0.304). Only one other miRNA (hsa-miR-148a-3p) correlated with serum glucose levels and also associated with serum glucose levels in the fully adjusted model 2 (Table 2, Supplementary Table S2).

**Table 2:** Significant (p_c_ < 0.05) associations between miRNAs and serum glucose and insulin levels, the HOMA2 insulin resistance index, and glycated hemoglobin (HbA1c) levels and percentage.

	Glucose		Insulin		HOMA2-IR		HbA1c		HbA1c %	
	p-value	r/β	p-value	r/β	p-value	r/β	p-value	r/β	p-value	r/β
**hsa-miR-144-5p**										
*Correlation*	**7**.67*10^−**7**^	**−0**.**167**	**1**.35*10^−**8**^	−**0**.**192**	**1**.81*10^−**8**^	−**0**.**190**	**8**.05*10^−**5**^	**−0**.**134**	**5**.80*10^−**5**^	**−0**.**137**
*Model 1*	**1**.57*10^−**7**^	**−0**.**163**	**8**.19*10^−**5**^	**−0**.**113**	**9**.99*10^−**5**^	**−0**.**111**	0.009	−0.086	0.009	−0.084
*Model 2*	**1**.22*10^−**5**^	**−0**.**14**								
**hsa-miR-148a-3p**										
*Correlation*	**1**.71*10^−**4**^	**−0**.**136**	0.033	−0.078	0.023	−0.083	**1**.39*10^−**5**^	**−0**.**158**	**9**.39*10^−**6**^	**−0**.**161**
*Model 1*	0.002	−0.103					**7**.72*10^−**5**^	**−0**.**135**	**6**.00*10^−**5**^	**−0**.**137**
*Model 2*	0.015	−0.082					**5**.05*10^−**5**^	**−0**.**14**	**3**.74*10^−**5**^	**−0**.**143**
**hsa-miR-122-5p**										
*Correlation*	3.17*10^−4^	0.135	**1**.49*10^−**11**^	**0**.**251**	**7**.37*10^−**12**^	**0**.**255**	3.58*10^−4^	0.134	2.23*10^−4^	0.139
*Model 1*			**1**.52*10^−**4**^	**0**.**119**	**6**.41*10^−**5**^	**0**.**124**	0.004	0.105	0.004	0.107
*Model 2*			2.48*10^−4^	0.109	2.09*10^−4^	0.110	0.005	0.104	0.005	0.104
**hsa-miR-184**										
*Correlation*	0.002	−0.108	**2**.86*10^−**5**^	**−0**.**145**	**1**.03*10^−**5**^	**−0**.**153**				
*Model 1*			0.001	−0.099	**1**.76*10^−**4**^	**−0**.**109**				
*Model 2*	0.007	−0.088	0.004	−0.08	0.003	−0.084				
**hsa-miR-339-3p**										
*Correlation*			**4**.44*10^−**5**^	**−0**.**140**	**4**.66*10^−**5**^	**−0**.**139**				
*Model 1*			0.001	−0.094	0.001	−0.095				
*Model 2*			0.006	−0.077	0.003	−0.083				
**hsa-miR-15b-3p**										
*Correlation*			0.006	−0.099	0.008	−0.096	0.001	−0.121	**1**.27*10^−**4**^	**−0**.**139**
*Model 1*			0.003	−0.089	0.007	−0.082	**1**.80*10^−**4**^	**−0**.**128**	**5**.55*10^−**5**^	**−0**.**138**
*Model 2*									0.031	−0.075
**hsa-miR-93-3p**										
*Correlation*					0.045	−0.068	**3**.29*10^−**6**^	**−0**.**157**	**5**.78*10^−**6**^	**−0**.**153**
*Model 1*			0.036	−0.060	0.019	−0.066	**8**.73*10^−**7**^	**−0**.**157**	**1**.11*10^−**6**^	**−0**.**155**
*Model 2*							**8**.21*10^−**6**^	**−0**.**145**	**1**.06*10^−**5**^	**−0**.**143**
**hsa-miR-146b-5p**										
*Correlation*			0.011	0.086	0.014	0.084	**3**.32*10^−**6**^	**0**.**157**	**2**.39*10^−**6**^	**0**.**159**
*Model 1*							**9**.57*10^−**5**^	**0**.**126**	**9**.39*10^−**5**^	**0**.**126**
*Model 2*							**8**.05*10^−**5**^	**0**.**123**	2.58*10^−4^	0.122
**hsa-miR-221-3p**										
*Correlation*			0.013	0.085	0.015	0.082	**9**.62*10^−**5**^	0.132	**2**.74*10^−**4**^	0.123
*Model 1*							**1**.82*10^−**4**^	0.120	0.001	0.109
*Model 2*							0.006	0.095	0.014	0.085
**hsa-miR-642a-5p**										
*Correlation*							**4**.51*10^−**7**^	**0**.**170**	**7**.95*10^−**7**^	**0**.**167**
*Model 1*							**8**.46*10^−**8**^	**0**.**171**	**1**.73*10^−**7**^	**0**.**167**
*Model 2*							**8**.70*10^−**8**^	**0**.**176**	**1**.54*10^−**7**^	**0**.**173**
**hsa-miR-181a-2-3p**										
*Correlation*							**2**.50*10^−**5**^	**0**.**143**	**2**.92*10^−**5**^	**0**.**142**
*Model 1*							**1**.62*10^−**4**^	**0**.**121**	2.10*10^−4^	0.119
*Model 2*							0.001	0.110	0.001	0.106
**hsa-miR-18a-3p**										
*Correlation*							**1**.30*10^−**4**^	**−0**.**130**	**1**.18*10^−**4**^	**−0**.**131**
*Model 1*							0.001	−0.109	0.001	−0.106
*Model 2*							0.002	−0.104	0.002	−0.102

Associations are evaluated with Spearman’s correlation and stepwise linear regression models. *Multiply sign. Only p-values smaller than 0.05 are shown here, and those with a p_c_ < 0.05 are indicated by bold font. All p-values, numbers of samples, and 95% CIs are shown in Supplementary Tables 2 and 3.

Statistical model: Correlation = Spearman correlation; Model 1 = Stepwise regression model including miRNA of interest (one by one), age, sex, and BMI; Model = Model 1 + leukocyte, erythrocyte, and thrombocyte count, in addition to total cholesterol, LDL, HDL, and triglyceride levels, as well as glycemic status, alcohol consumption, and history of smoking or hypertension.

Hsa-miR-122-5p, -184, and -339-3p correlated with insulin levels and HOMA2 index (p_c_ < 0.05). Hsa-miR-122-5p, which was also up-regulated in individuals with T2D, is the only miRNA with a direct correlation with these indicators of insulin resistance. Unlike hsa-miR-144-5p, these three miRNAs have an independent association with insulin levels and HOMA2 index in the fully adjusted model (p < 0.05) (Table 2, Supplementary Table S2).

Hsa-miR-144-5p, -148a-3p, -15b-3p, -93-3p, -146b-5p, -221-3p, -642a-5p, -181a-2-3p, and -18a-3p correlated with either HbA1c and/or HbA1c% (p_c_ < 0.05). Hsa-miR-148a-3p, -15-3p, -93-5p, and -18-3p had an inverse correlation with HbA1c/HbA1c%, while the other miRNAs had a positive correlation with their levels. All except hsa-miR-144-5p associated significantly with the levels of HbA1c/HbA1c% in the fully adjusted model (Table 2, Supplementary Table S3).

### Distinct miRNAs are associated with indicators of glucose levels and insulin resistance in NG and IFG individuals

In NG individuals, significant (p_c_ < 0.05) correlations were seen only between miRNA levels and levels of HbA1c or HbA1c%. Hsa-miR-221-3p and -642a-5p, which were associated with HbA1c and HbA1c% in the whole population, had a positive correlation with these variables, and these miRNAs had an independent association with HbA1c levels and percentages in the fully adjusted model as well. In addition, hsa-miR-589-3p, which was up-regulated in individuals with T2D, correlated negatively with HbA1c and HbA1c% and also associated with these values in the fully adjusted model. In individuals with IFG, hsa-miR-589-3p was not associated with HbA1c and HbA1c% levels, and in individuals with T2D (n = 24), the correlation was positive (p = 0.032, r = 0.438 and p = 0.034, r = 0.435 respectively), but this did not survive multiple testing correction (p_c_ > 0.05). Hsa-miR-454-5p had a correlation (p_c_ < 0.05) only with HbA1c%, even though it was associated with both HbA1c and HbA1c% (p_c_ < 0.05) in the fully adjusted model (Table 3 and Supplementary Tables S4 and S5).

**Table 3:** Significant (p_c_ < 0.05) associations between miRNAs and serum glucose and insulin levels, the HOMA2 insulin resistance index, and glycated hemoglobin (HbA1c) levels and percentage in normoglycemic individuals and prediabetics separately.

	Serum glucose		Insulin		HOMA2-IR		HbA1c		HbA1c %	
	p-value	r/β	p-value	r/β	p-value	r/β	p-value	r/β	p-value	r/β
**In normoglycemic individuals**										
hsa-miR-589-3p										
*Correlation*			0.007	−0.117	0.007	−0.117	**1**.80*10^−**5**^	**−0**.**185**	**4**.80*10^−**5**^	**−0**.**175**
*Model 1*			0.045	−0.077	0.049	−0.075	**4**.61*10^−**5**^	**−0**.**167**	**1**.37*10^−**4**^	**−0**.**154**
*Model 2*							**1**.03*10^−**4**^	**−0**.**158**	2.68*10^−4^	−0.147
hsa-miR-221-3p										
*Correlation*							**4**.50*10^−**5**^	**0**.**176**	**1**.29*10^−**4**^	**0**.**165**
*Model 1*							**4**.05*10^−**6**^	**0**.**189**	**7**.57*10^−**6**^	**0**.**181**
*Model 2*							**7**.47*10^−**5**^	**0**.**170**	**1**.15*10^−**4**^	**0**.**163**
hsa-miR-642a-5p										
*Correlation*							**7**.80*10^−**5**^	**0**.**170**	2.63*10^−4^	0.157
*Model 1*							**7**.20*10^−**6**^	**0**.**184**	**1**.91*10^−**5**^	**0**.**173**
*Model 2*							**2**.49*10^−**6**^	**0**.**192**	**5**.58*10^−**6**^	**0**.**183**
hsa-miR-454-5p										
*Correlation*							2.42*10^−4^	−0.174	**1**.34*10^−**4**^	**−0**.**181**
*Model 1*							**1**.54*10^−**4**^	**−0**.**170**	**9**.94*10^−**5**^	**−0**.**171**
*Model 2*							2.23*10^−4^	−0.165	**1**.40*10^−**4**^	**−0**.**167**
**In individuals with IFG**										
hsa-miR-885-5p										
*Correlation*	**9**.00*10^−**5**^	**0**.**244**	0.038	0.131	0.029	0.138				
*Model 1*	0.007	0.169	0.010	0.135	0.007	0.142				
*Model 2*	0.008	0.167	0.007	0.141	0.005	0.146				
hsa-miR-106b-5p										
*Correlation*	**9**.20*10^−**5**^	**0**.**244**	0.048	0.124	0.036	0.132				
*Model 1*	2.82*10^−4^	0.223					0.041	−0.127	0.043	−0.125
*Model 2*	2.09*10^−4^	0.230								
hsa-miR-122-5p										
*Correlation*			**8**.42*10^−**7**^	**0**.**334**	**9**.31*10^−**7**^	**0**.**333**				
*Model 1*			2.81*10^−4^	0.206	3.00*10^−4^	0.206				
*Model 2*			**1**.76*10^−**4**^	**0**.**207**	**2**.05*10^−**4**^	**0**.**206**				
hsa-miR-146b-5p										
*Correlation*							**4**.90*10^−**5**^	**0**.**254**	**6**.30*10^−**5**^	**0**.**250**
*Model 1*							**2**.56*10^−**5**^	**0**.**260**	**4**.41*10^−**5**^	**0**.**252**
*Model 2*							**6**.05*10^−**5**^	**0**.**251**	**5**.25*10^−**5**^	**0**.**252**

Associations are evaluated with Spearman’s correlation and stepwise regression models. *Multiply sign. Only p-values smaller than 0.05 are shown here, and those with a p_c_ < 0.05 are indicated by bold font. All p-values, numbers of samples, and 95%CIs are shown in Supplementary Tables 4 and 5.

Statistical model: Correlation = Spearman’s correlation; Model 1 = Stepwise regression model including miRNA of interest (one by one), age, sex, and BMI; Model 2 = Model 1 + leukocyte, erythrocyte, and thrombocyte count, in addition to total cholesterol, LDL, HDL, and triglyceride levels, as well as glycemic status, alcohol consumption, and history of smoking or hypertension.

In individuals with IFG, hsa-miR-122-5p and -146b-5p showed association patterns similar to their patterns in the whole population, with hsa-miR-122-5p levels having an independent association with insulin levels and HOMA2 index, while hsa-miR-146b-5p associated with the levels of HbA1c and HbA1c%. Furthermore, hsa-miR-885-5p and -106b-5p correlated positively with serum glucose levels and also had an independent association with glucose levels in the fully adjusted model in individuals with IFG (Table 3, Supplementary Tables S4 and S5).

### Expression patterns and technical validation of data

No clear expression clusters were identified when analyzing the miRNAs of interest. We could see an increased amount of significant positive correlations between miRNAs that associated with insulin levels/HOMA2-IR index and, similarly, between those that associated with HbA1c and HbA1c% levels, but the most significant correlations were the negative associations between hsa-miR-221-3p, and hsa-miR-589-3p and hsa-miR-18a-3p (Supplementary Fig. S3).

The functionality of the miRNA profiling arrays has been previously validated in a smaller sample population (n = 72) by correlating the results obtained by this method with those achieved with Human MiRNA Microarray Release 14.0, 8 × 15 K (Agilent). The correlation between the methods was good, and the association between hsa-miR-144-5p and serum glucose levels, for example, was also seen in the results obtained with the Agilent array^20^. To further validate the results, we detected a similar pattern in the expressions of hsa-miR-144-5p and -let-7a between the glycemic status groups (Supplementary Fig. S1) and even succeeded in replicating the nominally significant difference between NG and IFG groups in has-let-7a levels (p = 0.003, FC = 0.97) and the borderline significant result in hsa-miR-144-5p levels (p = 0.061, FC = 0.91) in the same setting.

### MicroRNAs hsa-miR-146b-5p, -221-3p and -589-3p associated with blood cell counts, while only hsa-miR-122-5p levels are originated solely from serum

To analyze the possibility that the miRNAs that were associated with the individuals’ glycemic status, glucose levels, or indicators of insulin resistance are expressed particularly in certain circulatory blood cells, we correlated the levels of these miRNAs with the leukocyte, erythrocyte, and thrombocyte counts. Out of the 16 miRNAs, hsa-miR-146b-5p and -589-3p correlated significantly with the leukocyte count (p = 1.01*10^−4^, r = 0.132 and p = 7.20*10^−5^, r = −0.135), and hsa-miR-589-3p hd an even stronger negative correlation with the erythrocyte count (p = 4.85*10^−6^, r = −0.155). Also, hsa-miR-106b-5p levels correlated with the leukocyte count in the whole population (p = 2.60*10^−5^, r = −0.143), but no association was seen in the subpopulation of individuals with IFG, where hsa-miR-106b-5p associated with glucose levels. Only levels of hsa-miR-221-3p correlated significantly with the thrombocyte count (p = 1.58*10^−18^, r = 0.293).

When repeating the non-parametric tests and correlation in the serum samples (n = 146) of YFS, only results regarding hsa-miR-122-5p were replicated, indicating that the levels of this miRNA originate solely from serum. Hsa-miR-184, -589-3p, and 18-a-3p were not sufficiently expressed in serum samples to be included in the analysis, and the rest of the miRNAs of interest did not show an association with glycemic status groups or indicators of glucose metabolism in serum. With hsa-miR-122-5p, the FC between the IFG and NG groups is substantially greater in serum samples (FC = 1.75, p = 0.025) (Supplementary Fig. S2) in comparison to whole blood (FC = 1.14, p = 0.006), indicating that the presence of various other blood components in varying amounts does contribute noise to the measurements. In line with these results, also the correlation coefficients between hsa-miR-122-5p and glucose, insulin, and HOMA2-IR were greater in serum samples (r = 0.353, p = 2.36*10^−5^; r = 0.412, p = 5.54*10^−7^ and r = 0.419, p = 3.56*10^−7^, respectively) in comparison to blood samples (Supplementary Table S8).

### Targets of hsa-miR-221-3p are enriched in pathways important to the development of T2D, while targets of hsa-miR-146b-5p are enriched in inflammatory pathways

The number of predicted targets with a correlation with a miRNA of interest varied greatly between the miRNAs (Supplementary File II, Correlation Tables 1–13). A correlation with a p_c_ < 0.05 were only seen with miRNAs whose levels differed significantly between the glycemic status groups (let-7a-5p and hsa-miR-589-3p) or those with a significant association with HbA1c and HbA1c% (hsa-miR-93-3p, -146b-5p, -148a-3p, -221-3p and -642a-5p). The greatest number of associations were found between hsa-miR-221-3p (111 correlations with p_c_ < 0.05) and hsa-miR-146b-5p (42 correlations with p_c_ < 0.05) and their targets. These were also the only miRNAs whose predicted targets were enriched in KEGG pathways (FDR q-value < 0.05) (Table 4). The insulin signaling pathway and Type 2 diabetes mellitus pathways were most significantly enriched by the targets of hsa-miR-221-3p and were selected for closer investigation. In addition, we could see enrichment of hsa-miR-146b-3p targets in inflammatory pathways and a pathway related to the cytoskeleton.

**Table 4:** Pathways enriched with the predicted targets of the miRNAs of interest.

Description	Genes in pathway	Genes in overlap	p-value	FDR q-value
**Hsa-miR-221-3p**				
Insulin signaling pathway	137	8	6.60*10^−5^	0.006
Type II diabetes mellitus	47	5	9.63*10^−5^	0.006
Glycosylphosphatidylinositol(GPI)-anchor biosynthesis	25	4	9.75*10^−5^	0.006
Alzheimer’s disease	169	8	2.81*10^–4^	0.012
Cysteine and methionine metabolism	34	4	3.33*10^−4^	0.012
Tight junction	134	7	3.72*10^−4^	0.012
Purine metabolism	159	7	0.001	0.027
Spliceosome	128	6	0.002	0.039
**Hsa-miR-146b-5p**				
Toll-like receptor signaling pathway	102	6	1.04*10^−4^	0.017
Adherens junction	75	5	2.21*10^−4^	0.017
Peroxisome	78	5	2.65*10^−4^	0.017
Regulation of actin cytoskeleton	216	7	0.001	0.045
Epithelial cell signaling in Helicobacter pylori infection	68	4	0.002	0.045
Pancreatic cancer	70	4	0.002	0.045
Leishmania infection	72	4	0.002	0.045
Lysosome	121	5	0.002	0.045
Endocytosis	183	6	0.002	0.046
Spliceosome	128	5	0.002	0.046

Only targets that were predicted by two algorithms and whose expression levels correlated with miRNA levels (p < 0.05) were included in the enrichment analysis. Significant results (FDR q-value < 0.05) were found only with hsa-miR-221-2p and -146b-5p.

Abbreviations: FDR = false discovery rate. *Multiply sign.

### MicroRNAs that associate with HbA1c levels are associated with the levels of genes in the insulin signaling and T2D pathway

**Figure 2:**
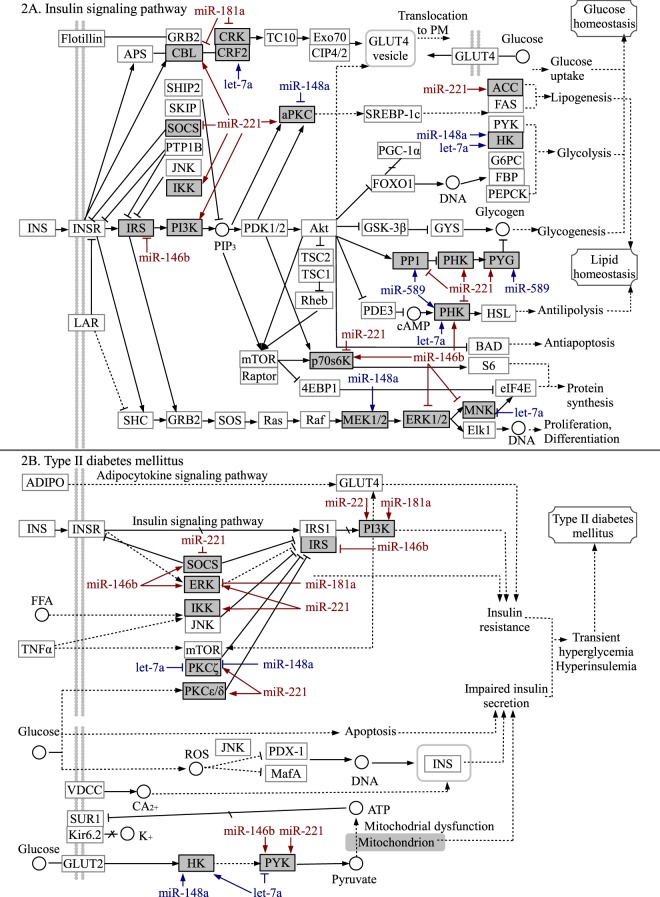
Association between miRNAs of interest and their predicted targets in the insulin signaling pathway (**A**) and Type II diabetes mellitus pathway (**B**)^52^. Transcripts whose expression correlated significantly (p < 0.05) with the miRNAs of interest and whose levels were independently and significantly associated with the targeting miRNA in the fully adjusted regression model* are marked with grey boxes. MicroRNAs whose expression correlated positively with glucose/insulin/HbA1c are indicated in red, while those with a negative correlation or down-regulation in individuals with IFG in comparison to NG are indicated in blue. Positive correlation between miRNA and its target mRNA is marked with  and negative correlation with . *Statistical model: Stepwise AIC linear regression model including the miRNA of interest, age, sex, BMI, leukocyte count, erythrocyte count, thrombocyte count, glycemic status, glucose insulin, as well as HbA1c levels, HbA1c%, and HOMA2 IR index statistically predicting the mRNA target.

To assess the possible co-regulation of the miRNAs of interest in the insulin signaling pathway and Type 2 diabetes signaling pathway we correlated the genes in these pathways with the miRNAs that were predicted to target them. Our results show that the HbA1c-associated miR-181a-2-3p, -146b-5p, -148a-3p, and -221-3p, and hsa-let-7a and miR-589-3p, were also independently and significantly (p < 0.05) associated with mRNA levels of the insulin signaling pathway and type 2 diabetes pathway genes (Fig. 2A,B, Supplementary Tables S6 and S7). Most interestingly, hsa-miR-146b-5p correlated highly significantly and negatively with 5′-AMP-activated protein kinase subunit gamma-2 (PRKAG2) mRNA levels, indicating the possible repression of mRNA target. We could also see a similar association between hsa-miR-221-3p and protein phosphatase 1 catalytic subunit beta (PPP1CB) and ribosomal protein S6 kinase B1 (RPS6KB1).

## Discussion

T2D is a complex and heterogenic multi-organ disease, which is preceded by a state of increased blood glucose and the development of insulin resistance. We show here, in a general-population-based cohort, that IFG changes are associated with the miRNA expression in whole blood and that elevated levels of serum glucose, insulin, and glycated hemoglobin are strongly associated with the miRNA levels. Gene expression data from the same individuals indicates that miRNAs whose expression is associated with HbA1c may also regulate the expression of their targets in the insulin signaling and type 2 diabetes mellitus pathway.

In YFS, the blood levels of hsa-miR-144-5p and hsa-let-7a-5p were down-regulated in individuals with prediabetes. We have previously reported a negative correlation between whole blood hsa-miR-144-5p and serum glucose levels in a pilot population of YFS^20^ and now show herein that this miRNA is associated with (with negative β value) serum glucose, insulin, and HOMA2 index levels in the fully adjusted model. In contrast to our results, miR-144-5p has been previously reported to be up-regulated in the blood and blood fractions of diabetics^21–23^. In the regulation of glucose homeostasis, hsa-miR-144-5p has been shown to directly target Insulin receptor substrate 1 (IRS1)^23^ and Glucose transporter GLUT1^24^ and thus regulate glucose metabolism on many levels. In our whole blood samples, we saw a strong association between hsa-miR-144-5p and serum triglyceride levels, possibly indicating that this miRNA may be connected to the development of T2D also through a role in fatty acid homeostasis, but no significant correlation with target mRNAs was observed. Hsa-let-7a-5p has also been associated with T2D. Its levels in exosomes have been shown to be decreased in individuals with T2D, and, interestingly, the levels increased after 12 months of diabetic medication^25^. In our transcriptomic analysis, hsa-let-7a-5p levels were associated with the levels of its predicted targets in pathways leading to glycolysis and mitochondrial dysfunction.

Hsa-miR-122-5p was significantly up-regulated in individuals with T2D, and the levels of this miRNA also correlated positively with insulin levels and the HOMA2-IR index in both the whole population and individuals with IFG. We and others have shown that the circulatory levels of this miRNA are up-regulated in whole blood and the blood fractions when fatty liver develops^26,27^. As up to 90% of diabetic individuals have some degree of fatty liver, it is reasonable to assume that the increase in this miRNA in our population reflects the development of fatty liver concomitantly with the dysregulation of glucose homeostasis. Although the levels of hsa-miR-122-5p have been measured from the whole blood samples of the larger YFS population, our results from the serum of the subpopulation of YFS participants indicates that the signal mainly comes from the serum. Hsa-miR-885-5p levels were also shown to be up-regulated in individuals with fatty liver in the YFS^26^, and in the IFG subpopulation we can see that this miRNA also positively correlates with glucose levels, indicating a similar expression pattern to that of hsa-miR-122-5p.

Levels of hsa-miR-589-3p, a miRNA up-regulated in the whole blood of individuals with T2D, also correlated negatively with HbA1c levels in NG individuals. In the T2D populations, the correlation was positive, although it was not significant enough to survive the multiple testing correction and no correlation was seen in the IFG population. In our transcriptomics analysis, hsa-miR-589-3p levels correlated positively with transcripts inhibiting glycogenesis in the type 2 diabetes pathway. This may partly explain the complicated expression pattern of hsa-miR-589-3p in our population, as high blood glucose activates glycogenesis in individuals with a functional regulation of glucose levels, while defective glycogenesis is involved in the worsening of glucose level regulation in T2D^28^.

Hsa-miR-184 blood levels correlated negatively with insulin and HOMA2-IR index. This miRNA has been previously shown to be pancreas-enriched, and its expression has been shown to correlate negatively with glucose-stimulated insulin secretion^29^. It has been shown to regulate insulin secretion in a cell culture model^30^ and in the compensatory β-cell proliferation and secretion during insulin resistance in mice^31^. The expression of hsa-miR-184 has been shown to be increased in the pancreatic islets of mice after fasting and to be down-regulated after the administration of a sucrose rich diet in drosophila^31^. No previous report exists on the correlation between circulatory levels of hsa-miR-184 and serum insulin levels, but our results indicate that the pancreatic down-regulation of this miRNA in the development of peripheral insulin resistance and the requirement of increased levels of insulin can also be seen in whole blood. In addition to hsa-miR-184, hsa-miR-339-3p levels correlated with insulin levels and the HOMA2-IR index. This miRNA has also been associated with the development of pancreatic islets^32^ and it has been reported to regulate the expression of glucose-6-phospahatse, the enzyme catalyzing the final steps of gluconeogenesis and glycogenolysis^33^, suggesting potential participation in the development of insulin resistance.

A total of eight miRNAs correlated with HbA1c levels/percentages and had an independent association in the fully adjusted model in the whole population. In addition, hsa-miR-454-5p had an association with HbA1c% in the NG subpopulation. Out of the combined nine miRNAs, miR-148a^16^ and -181a^34^ have been shown to be up-regulated in plasma and serum miR-15b^35^ and -18a^36^ to be down-regulated in T2D, while plasma miR-93^15^ and -148a^16^ have been shown to be down-regulated in prediabetics in comparison to healthy controls (Supplementary Table S9). Similar patterns of up-regulation of serum miR-148a and -181a^37^ and down-regulation of miR-93^38^ in PBMCs have been reported in type 1 diabetes. The associations between these miRNAs and HbA1c levels/percentages are well in line with their previously reported directions of regulation in T2D and prediabetes. Several of these miRNAs (miR-148^39^, -93^40^ and -146^41^) have been associated with obesity or/and the differentiation or phenotype of the adipocytes. In addition, miR-221 and -146 are known to be associated with inflammation^42^, and we were able to show that hsa-miR-146b-5p levels correlated with the leucocyte count, indicating that, in our samples, this miRNA may originate from inflammatory cells. In addition, in our data the predicted target mRNAs of hsa-miR-146b-5p were enriched in several inflammatory pathways—for example, the Toll-like receptor signaling pathway. Interestingly, miR-15b has been shown to be down-regulated in the skeletal muscles of twins with T2D in comparison to those without T2D^43^, to suppress pancreatic β-cell proliferation and insulin secretion, and to possibly convey the effects of intrauterine conditions into later life in mice^44^.

We detected a negative association between hsa-miR-93-3p and HbA1c levels. The expression of this miRNA has been shown to be down-regulated by high glucose in podocytes^45^ and in the plasma of prediabetic individuals^46^. It has also been shown to regulate the expression of Glucose transporter type 4 (GLUT4), the main glucose transporter in peripheral tissues, and of vascular endothelial growth factor (VEGF), which has an important role in the microvascular complications of diabetes; but it has also been shown to have a role in atherosclerosis. In addition, hsa-miR-221-3p, which associated with HbA1c levels, has been shown to be regulated by glucose levels, to mediate endothelial dysfunction^47,48^, and to be elevated in the internal thoracic arteries of individuals with T2D, but these levels were normalized by the usage of Metformin^49^. The predicted targets of this inflammation-related miRNA^42^ were seen to be enriched in the insulin signaling and type II diabetes mellitus pathways. Most interestingly, a negative correlation was seen between hsa-miR-221-3p levels and levels of RPS6KB1 (p70S6K), which has been associated with insulin sensitivity, aging, and obesity, and serine/threonine-protein phosphatase PP1-beta catalytic subunit levels, which are known to participate in glycogen metabolism. Hsa-miR-221-3p levels also significantly correlated with the thrombocyte count, suggesting a possible cell type of origin. Our results thus indicate that hsa-miR-93-3p and -221-3p could mediate the effects of elevated blood glucose to the vascular endothelial cells.

When analyzing the co-regulation of the miRNAs of interest in the insulin signaling pathway and Type 2 diabetes mellitus pathway, we were able to see a significant correlation between hsa-miR-146-5p and its predicted target PRKAG2, a part of the AMP-activated protein kinase and a major cellular regulator of lipid and glucose metabolism. Hsa-miR-181a-2-3p levels, on the other hand, correlated negatively with genes leading to the transport of GLUT4 to the plasma membrane in the insulin signaling pathway, while it seemed to activate the route leading to insulin resistance in the type II diabetes mellitus pathway. Transcriptomic analysis also showed a significant association between miR-148a and its predicted target heksokinase 1, the enzyme catalyzing the first step of glycolysis, which has previously been shown to also be associated with the levels of HbA1c^50^.

A limitation of our study is that profiling miRNAs from blood poses a challenge for identifying the origin of the miRNAs, as blood contains miRNAs from circulatory cells but also circulatory miRNA originating from various tissues^26^. As the whole blood levels can be affected by the amount of exportation from surrounding tissues, but also by the changes in in expression in circulatory cells, the whole blood miRNA levels are not necessarily representative of the miRNA levels in tissues important to development of T2D, such as the pancreas or skeletal muscle. Our results with hsa-miR-122-5p do indicate that, if the miRNA levels originate solely from serum, analyzing the miRNA levels from whole blood increases the noise in the measurement and reduces the magnitude of the FCs and correlation coefficients. Whole blood was selected to enable gene-expression analysis from the same sample^26^. As almost all of the mRNAs quantified from whole blood originate from circulatory blood cells, our target analysis mainly represents gene expression regulation in hematopoietic cells and may not fully describe the processes in other tissues important to T2D. Oral glucose tolerance tests had not been performed for the study population, and hence we are unable to reflect upon the association between blood miRNA profiles and impaired glucose tolerance. In addition, replication in other populations is needed to verify the findings. The YFS population is still rather young and just approaching the age where T2D is most frequently diagnosed. Because of this, we can provide information about the miRNAs’ associations with physiological glucose levels, but as the number of individuals with T2D is low, further analysis is needed to connect the discovered miRNAs to the onset of T2D. The strengths of this study are the large, well-phenotyped population-based cohort and the availability of genome-wide gene expression data from the same samples, which enables detailed analysis of specific biological processes through which miRNAs may exert their effects. As this work is essentially descriptive, more research is needed to shed light on the mechanism of how miRNAs are released into the blood and to validate the interaction of the discovered miRNAs and their targets^26^. In addition, as T2D is a heterogenous disease with different progression patters, it is possible that known and unknown cofactors are affecting our results.

In conclusion, we are able to show that, in a population-based study cohort, glycemic status and blood glucose/insulin/HbA1c levels are associated with the miRNA expression profile in whole blood. These associations are well in line with previous results from peripheral tissues and the pancreas during the development of T2D. There were also significant differences in the blood miRNA profiles associating with serum glucose, insulin, or IR levels when compared to those associating with HbA1c. As the HbA1c-associated miRNAs were strongly associated with the gene expression of the target mRNAs in insulin signaling and type II diabetes mellitus pathways, it can be hypothesized that long-term glucose levels in particular can affect gene expression via miRNA regulation.

## Methods

### See the full research design and methods information in the supplementary materials

#### The Young Finns Study (YFS)

YFS is a multi-center follow-up study on cardiovascular risk from childhood to adulthood in Finland. The YFS was launched in 1980, with 3,596 children and adolescents randomly selected from the Finnish national population register. The 30-year follow-up was performed in 2011, with 2,063 adults aged 34–49 years participating in the study. The examinations included physical measurements, blood tests, and questionnaires^51^. The present study has been approved by the 1st ethical committee of the Hospital District of Southwest Finland on September 21st, 2010 and by local ethical committees. All study subjects gave an informed consent, and the study was conducted according to the principles of the Declaration of Helsinki. As previously described^26^, the YFS samples for miRNA analysis (n = 992) were selected independently of glycemic status from individuals with the most comprehensive data. After quality control, the study population comprises 871 individuals with successful miRNA profiling (Table 5).

**Table 5:** Demographics of the Young Finns Study participants with successful miRNA profiling.

	All*	NG	IFG	T2D
Number of subjects	871	534	252	24
Age, years	43 (4.8)	41.9 (4.9)	43.2 (4.7)	45.1 (3.5)
Males, (%)	45.4	38.8	59.9	54.2
Total cholesterol, mmol/l	5.1 (0.9)	5.1 (0.9)	5.3 (0.9)	5.0 (0.9)
HDL cholesterol, mmol/l	1.3 (0.4)	1.4 (0.3)	1.3 (0.3)	1.2 (0.4)
LDL cholesterol, mmol/l	3.2 (0.8)	3.2 (0.8)	3.4 (0.9)	3.0 (0.8)
Triglycerides, mmol/l	1.1 (0.7)	1.1 (0.7)	1.4 (0.7)	1.8 (0.9)
Medication for hypercholesterolemia (%)	3.4	1.9	4.8	12.5
Blood glucose, mmol/l	5.3 (0.9)	5.1 (0.3)	5.7 (0.5)	7.0 (1.8)
HbA1c, %,	5.4 (0.4)	5.4 (0.2)	5.6 (0.3)	6.6 (1.0)
HbA1c, mmol/mol	36.0 (4.6)	25.2.0)	38.1 (3.0)	48.1 (11.3)
Insulin, mU/l	9.9 (15.4)	7.0 (4.9)	11.4 (8.8)	19.4 (14.6)
HOMA2 IR	1.1 (2.1)	0.9 (0.6)	1.5 (1.1)	2.6 (2.0)
Oral diabetes medication (%)	1.0	0	0.4	33.3
Insulin medication (%)	0.3	0	1.2	0
Hypertension, (%)	8.2	5.2	11.1	37.5
Systolic, mmHg	117.7 (13.9)	116.3	123.7 (13.5)	126.1 (17.8)
Diastolic, mmHg	74.2 (10.5)	73.0 (10.0)	78.4 (10.2)	79.0 (9.1)
Medication for hypertension (%)	9.0	5.8	12.3	33.3
Body mass index, kg/m2	25.6 (4.9)	25.3 (3.9)	28.2 (5.5)	30.7 (6.7)
Fatty liver, (%)	3.2	0.9	5.6	20.8
Mild fatty liver, (%)	13.7	7.5	21.4	41.7
Leukocyte count, 10^9^/l	5.5 (1.5)	5.3 (1.3)	5.7 (1.5)	6.4 (2.2)
Erythrocyte count, 10^12^/l	4.7 (0.4)	4.6 (0.4)	4.8 (0.4)	4.7 (0.4)
Thrombocyte count, 10^9^/l	256.2 (58.1)	252.9 (58.9)	260 (52.8)	279.0 (54.9)

Continuous variables are presented by means, with standard deviations in parentheses.

*Individuals with type 1 diabetes or lacking information on blood glucose levels and/or HbA1c levels are not included in the groups determined by glycogenic status.

#### Clinical and biochemical measurements

As previously described^26^, weight, height, waist circumference, and blood pressure were measured, and body mass index (BMI) was calculated. Blood cell parameters were measured with flow cytometric particle counting and photometry. The serum triglyceride, glucose, and total cholesterol concentrations were analyzed using the enzymatic methods. HDL cholesterol levels were estimated after the precipitation of low-density lipoprotein (LDL) and very-low-density lipoprotein. For HbA1c fraction measurement, the concentration of total hemoglobin was determined colorimetrically, after which the concentration of HbA1c was measured immunoturbidimetrically. These two concentrations were used to calculate the HbA1c percentage (HbA1c%). Insulin levels were measured by a microparticle enzyme immunoassay kit, and the HOMA2 index was calculated according to the online HOMA2-IR calculator (https://www.dtu.ox.ac.uk/homacalculator/). Individuals were categorized into the normoglycemic (NG), IFG, and T2D groups. The classification of IFG was based on fasting serum glucose and HbA1c according to the criteria of the WHO^3^. Individuals with type 1 diabetes were discarded from the analysis.

#### Whole blood RNA isolation and miRNA expression profiling

The sample collection and miRNA profiling has been described previously^26^. In brief, whole blood was collected into PaXgene Blood RNA Tubes and RNA isolated with a PAXgene Blood MicroRNA Kit. MicroRNA expression profiling was performed with the TaqMan® OpenArray® MicroRNA Panel containing 758 microRNAs. Primary data analysis was performed with Expression Suite Software version 1.0.1. RNU6, RNU44, and RNU48 were used as housekeeping small RNAs. Two hundred and forty-three (243) miRNAs that were expressed in at least 2/3 of the samples were included in the analysis. The RNA quality and functionality of the panels has been validated previously^20^. Profiling was successful in 871 samples. To correct for batch effects, the principal component analysis was performed for the miRNA expression data.

#### Genome-wide expression analysis (transcriptomics)

The expression levels were analyzed with an Illumina HumanHT-12 version 4 Expression BeadChip^26^. Raw Illumina probe data was exported from Genomestudio and analyzed in R using the Bioconductor packages. The expression data was processed using nonparametric background correction, followed by quantile normalization with control and expression probes. The expression analysis was successful in 743 of the 871 samples with a miRNA expression profile.

#### Statistical analysis

MicroRNA expression differences over glycemic status groups were analyzed with Kruskal-Wallis analysis of variance, and the different glycemic status groups were then compared by using the Mann-Whitney U test. Bonferroni-corrected p-values (p_c_-value = nominal p-value/243) were calculated, and a p_c_-value of <0.05 (=p < 0.00021) was considered significant. For dysregulated miRNAs, fold changes (FCs) were calculated for each individual sample in comparison to the median of all NG individuals. To analyze whether dysregulated miRNAs were independent statistical predictors of IFG or T2D, a stepwise Akaike information criterion (AIC) logistic regression model was utilized. Two different models were used as follows: Model 1: The dependent variable was glycemic status as statistically predicted with the discovered miRNAs, age, sex, and BMI; and Model 2: Model 1 + leukocyte, erythrocyte, thrombocyte count, total cholesterol, LDL, HDL, and triglyceride levels, as well as alcohol consumption and history of smoking and hypertension. In the regression models, all continuous variables were inverse-normalized. The number of samples in the regression models varies according to the number of samples in which the miRNA was expressed and according to the availability of the variables in the regression models, as all measurements were not successful/available from all of the samples.

The associations between miRNA levels and glucose, insulin, HbA1c, HbA1c%, and HOMA2 IR index were correlated with Spearman’s rank-order correlation. P_c_-value < 0.05 was considered significant. The independent statistical prediction value was evaluated with AIC linear regression models with the same covariates as in the logistic regression models (Model 1 and Model 2), but with glycemic status also included in Model 2. Analyses stratified by glycemic status were performed as in the whole population, without glycemic status as a cofactor in Model 2. In regression models, p < 0.05 was considered significant.

To analyze the co-regulation of the miRNAs of interest and the existence of possible expression clusters of these miRNAs, between-miRNA Spearman’s rank-order correlations were analyzed. Also, to validate our results, we analyzed the differences in hsa-miR-144-5p and -let-7a-5p over the glycemic status groups with miRNA data profiled with Human MiRNA Microarray Release 14.0, 8 × 15 K (Agilent) from the whole blood of 72 individuals from the YFS 2011 follow-up. The sample preparation, miRNA profiling, and data preprocessing have been described previously^20^. Differences in microRNA expressions between glycemic status groups were analyzed, as with the larger data set, from whole blood, and hsa-miR-144-5p and -let-7a were selected because they were available for both arrays and gave significant results in the non-parametric tests.

To analyze the possibility that miRNAs associated with the individuals’ glycemic status, glucose levels, or indicators of insulin resistance are expressed particularly in certain circulatory blood cells, we correlated the levels of these miRNAs with the leukocyte, erythrocyte, and thrombocyte counts by means of Spearman’s rank-order correlation. In addition, we investigated whether some of the miRNAs were originating solely from serum by replicating the non-parametric tests, and the correlation between the miRNAs of interest and glycemic status, and the indicators of glucose metabolism in the serum miRNA profilings was performed with TaqMan® OpenArray® MicroRNA Panels from a subpopulation (n = 146) of the YFS (see supplementary materials and methods).

In the target mRNA analysis, Spearman’s rank-order correlations were performed between the FCs of the miRNAs of interest (miRNAs with significantly different levels between glycemic status groups or significant correlation with glucose, insulin, HbA1c, HbA1c% levels, or HOMA2 index in the whole population) and the expression of their predicted targets (predicted by two or more algorithms according to miRGator v.3.0, http://mirgator.kobic.re.kr/). Transcripts with a correlation p < 0.05 were included in a pathway enrichment analysis performed for the KEGG pathways in the molecular signatures database (http://software.broadinstitute.org/gsea/msigdb/index.jsp). Pathways with an FDR-q value of <0.05 were considered significantly enriched with target mRNAs. The most significant pathways were further analyzed for the coregulation of all of the miRNAs of interest. All predicted targets (predicted by at least one algorithm in miRGator 3.0) from at least one of the miRNAs of interest were selected from the most significantly enriched pathways and correlated (Spearman’s rank-order correlation) with their predicted regulatory miRNA levels. The predicted target mRNAs with significant correlation (p < 0.05) were included in further analysis. The independent statistical prediction values of the miRNAs of interest on the levels of target mRNA were evaluated with an AIC linear regression model including the miRNA of interest, age, sex, BMI, leukocyte count, erythrocyte count, thrombocyte count, glycemic status, as well as the glucose, insulin, and HbA1c levels, and HbA1c% and HOMA2 IR index. Transcripts whose levels were significantly (p < 0.05) and independently statistically predicted by the levels of the miRNAs of interest were considered to be affected by these miRNAs.

### Perspectives

As the prevalence of type 2 diabetes increases, it is crucial to understand the underlying molecular mechanisms. We found that there were significant differences in the blood microRNA profiles associated with serum glucose, insulin levels, or insulin resistance index compared to those associated with HbA1c. The HbA1c-associated miRNAs were strong statistical predictors of the expression of target mRNAs in pathways important to the development of T2D, highlighting the role of microRNAs in regulating pathways during T2D pathogenesis.

## Supplementary information

Supplementary material

Supplementary data

## Data Availability

The data sets generated and/or analyzed during the current study are not publicly available due to restrictions imposed by Finnish legislation but are available from the corresponding author upon a reasonable request.
