# Supplementary material for: Whole blood microRNA levels associate with glycemic status and correlate with target mRNAs in pathways important to type 2 diabetes
Source: Sci Rep. 2019 Jun 20;9:8887. doi: 10.1038/s41598-019-43793-4 (PMC6586838; doi:10.1038/s41598-019-43793-4)
Supplement: Supplementary file 1 — Supplementary material [file 41598_2019_43793_MOESM1_ESM.pdf]

Supplementary material

Whole blood microRNA levels associate with glycemic status and correlate with target mRNAs in pathways important to type 2 diabetes

Nina Mononen, PhD; Leo-Pekka Lyytikäinen, MD; Ilkka Seppälä, MD, PhD; Pashupati P Mishra, MSc; Markus Juonala, MD, PhD: Melanie Waldenberger, PhD; Norman Klopp, PhD; Thomas Illig, MD, PhD; Jaana Leiviskä, PhD; Britt-Marie Loo, PhD, Reijo Laaksonen, MD, PhD; Niku Oksala, MD, PhD, DSc; Mika Kähönen, MD, PhD; Nina Hutri-Kähönen, MD, PhD; Olli Raitakari, MD, PhD; Terho Lehtimäki, MD, PhD, Emma Raitoharju, PhD

Table of Contents

Supplementary Figures 2
  Supplementary Figure S1. 2
  Supplementary Figure S2. 3
  Supplementary Figure S3. 4
Supplementary Tables 5
  Supplementary Table S1 5
  Supplementary Table S2 6
  Supplementary Table S3 8
  Supplementary Table S4 9
  Supplementary Table S5 10
  Supplementary Table S6 11
  Supplementary Table S7 12
  Supplementary Table S8 13
  Supplementary Table S9 14
Supplementary Materials and Methods 15
  The Young Finns Study (YFS) 15
  Clinical and biochemical measurements 15
  Categorizing individuals according to T2D and glycemic status 16
  Whole blood RNA isolation, quality control, and miRNA expression profiling 16
  Serum RNA isolation and miRNA expression profiling 16
  Genome-wide expression analysis (transcriptomics) 17
  Statistical analysis 17
Supplementary references 19

Supplementary Figures

Supplementary Figure S1. Blood levels of hsa-miR-144-5p and hsa-let-7a-5p in normoglycemic individuals (NG), individuals with impaired fasting glucose (IFG), and individuals with type 2 diabetes (T2D), analyzed from the whole blood of a subset (n=72) of the YFS 2011 follow-up with Human MiRNA Microarray Release 14.0, 8 × 15 K (Agilent). The trend over groups is analyzed using the Kruskal–Wallis test (dash line) and the differences between groups by the Mann-Whitney U test (solid line). We can see a similar pattern of expression in hsa-miR-144-5p and hsa-let-7a-5p between the glycemic status groups to the pattern shown with the larger population analyzed with TaqMan Openarray and were even able to replicate the nominally significant difference between the NG and IFG groups in has-let-7a-5p levels (p=0.003) and the borderline significant result in hsa-miR-144-5p levels (p=0.061).

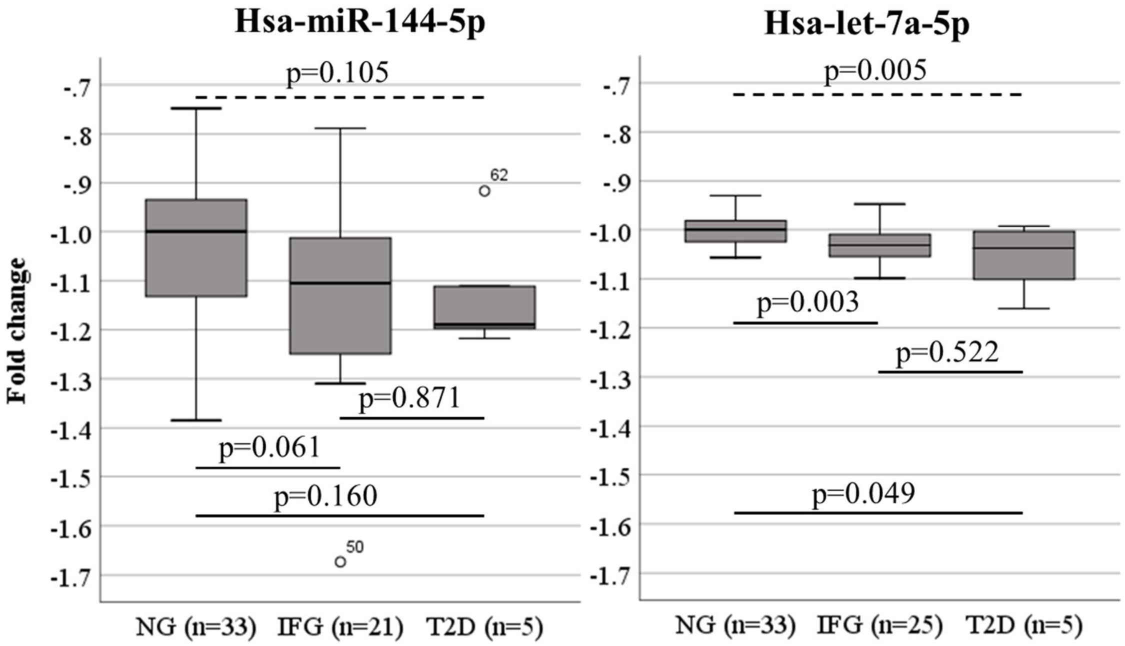

Supplementary Figure S2. Serum levels of hsa-miR-122-5p in normoglycemic individuals (NG), individuals with impaired fasting glucose (IFG), and individuals with type 2 diabetes (T2D), analyzed from the serum of a subset (n=146) of the YFS 2011 follow-up with the TaqMan® OpenArray® MicroRNA Panel. The trend over the groups is analyzed using the Kruskal–Wallis test (dash line) and the differences between the groups by the Mann-Whitney U test (solid line). We can see a similar pattern of expression in hsa-miR-122-5p between the glycemic status groups to the one shown with the larger population analyzed from whole blood and were even able to replicate the nominally significant difference between the NG and IFG groups and the trend over the glycemic status groups.

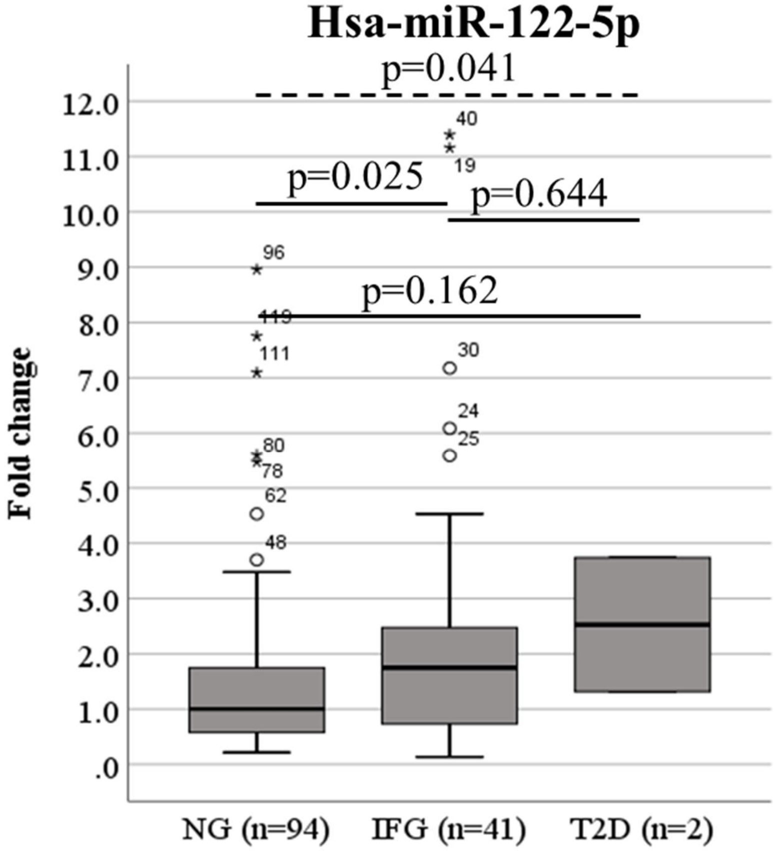

Supplementary Figure S3. Correlation plot between the miRNAs of interest in whole blood. Associations were evaluated with Spearman's rank-order correlation, and only associations with a nominal p<0.05 are shown with a circle.

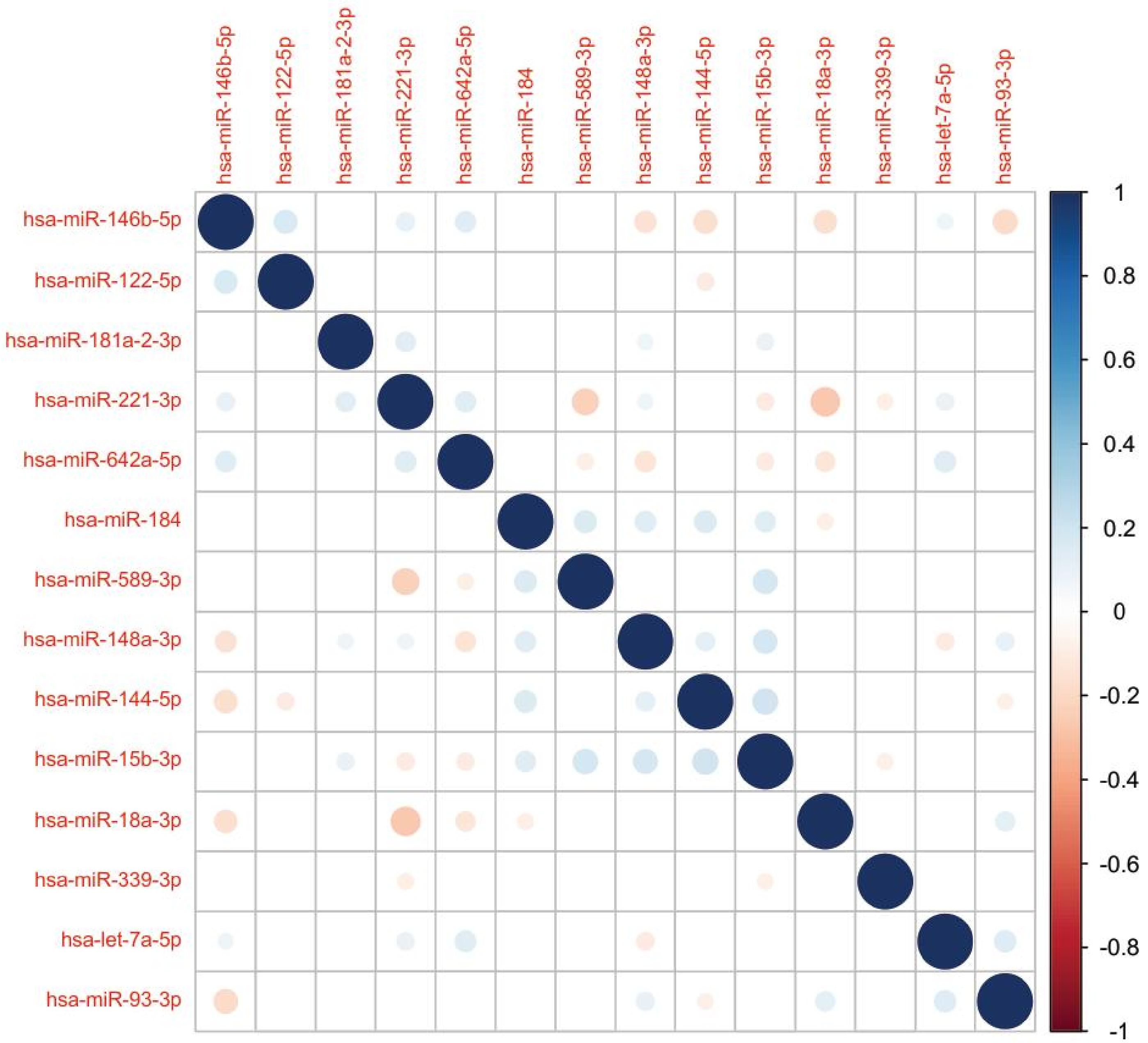

Supplementary Tables

Supplementary Table S1. Associations between miRNAs and the individuals' glycemic status (normoglycemic [NG]/ impaired fasting glucose [IFG]/type 2 diabetes [T2D]). Associations were evaluated with the Mann-Whitney U test and stepwise logistic regression models. Fold changes (FC) describe the magnitude of the difference with the Mann-Whitney U test, while odds ratios (OR) are shown with regression models.

	**IFG vs NG**			**T2D vs NG**			**T2D vs IFG**		
	**n**	**p**	**FC/OR (95% CI)**	**n**	**p**	**FC/OR (95% CI)**	**n**	**p**	**FC/OR (95% CI)**
hsa-miR-144-5p									
U-test	779	2.35E-06	0.91	553	0.033	0.90	274	0.761	0.99
Model 1	776	7.42E-05	0.71 (0.60-0.84)	551	0.043	0.63 (0.40-0.98)	273	NS	NS
Model 2	746	3.64E-04	0.73 (0.61-0.87)	532	NS	NS	260	NS	NS
hsa-let-7a-5p									
U-test	781	6.39E-06	-1.08	556	0.823	0.98	273	0.145	1.08
Model 1	778	3.00E-04	0.74 (0.62-0.87)	554	NS	NS	272	NS	NS
Model 2	747	4.50E-04	0.73 (0.61-0.87)	535	NS	NS	258	NS	NS
hsa-miR-122-5p									
U-test	634	0.006	1.14	446	4.80E-05	1.53	226	0.009	1.34
Model 1	632	0.146	1.14 (0.95-1.38)	445	0.002	2.68 (1.47-5.16)	225	0.042	1.64 (1.02-2.70)
Model 2	611	NS	NS	433	0.010	2.48 (1.29-5.22)	214	0.062	1.68 (0.99-2.98)
hsa-miR-589-3p									
U-test	781	0.149	-1.03	555	1.82E-04	1.29	274	6.60E-05	1.32
Model 1	778	NS	NS	553	2.34E-04	2.53 (1.57-4.24)	273	2.27E-04	2.51 (1.57-4.21)
Model 2	747	NS	NS	534	3.70E-04	2.80 (1.63-5.11)	259	1.65E-04	2.83 (1.70-5.05)

Statistical model: U-test = Mann-Whitney U test; Model 1 = Stepwise logistic regression model including an miRNA of interest (one by one), age, sex, and BMI; Model 2 = Model 1 + leukocyte, erythrocyte, and thrombocyte counts, total cholesterol, LDL, HDL, and triglyceride levels, as well as alcohol consumption and history of smoking or hypertension. **Note:** The number of samples in the regression models varies according to the number of samples in which the miRNA was expressed and also according to the availability of the variables in the regression models.

Supplementary Table S2. Associations of the miRNAs with serum glucose and insulin levels and the HOMA2 insulin resistance (IR) index. Associations were evaluated with Spearman’s correlation and stepwise linear regression models*.

	Serum glucose			Serum insulin levels			HOMA2-IR		
	**n**	**p**	**r/β (95% CI)**	**n**	**p**	**r/β (95% CI)**	**n**	**p**	**r/β (95% CI)**
hsa-miR-144-5p									
Correlation	863	7.67E-07	-0.167	863	1.35E-08	-0.192	860	1.81E-08	-0.190
Model 1	860	1.57E-07	-0.163 (-0.224– -0.103)	806	8.19E-05	-0.113 (-0.169– -0.057)	857	9.99E-05	-0.111 (-0.167– -0.055)
Model 2	769	1.22E-05	-0.140 (-0.203– -0.078)	769	NS	NS	769	NS	NS
hsa-miR-148a-3p									
Correlation	754	1.71E-04	-0.136	754	0.033	-0.078	752	0.023	-0.083
Model 1	753	0.002	-0.103 (-0.168 – -0.038)	753	NS	NS	751	NS	NS
Model 2	674	0.015	-0.082 (-0.148 – -0.016)	674	NS	NS	674	NS	NS
hsa-miR-122-5p									
Correlation	703	3.17E-04	0.135	703	1.49E-11	0.251	700	7.37E-12	0.255
Model 1	701	0.125	0.053 (-0.015–0.120)	701	1.52E-04	0.119 (0.058–0.180)	698	6.41E-05	0.124 (0.064–0.185)
Model 2	629	0.114	0.055 (-0.013–0.122)	629	2.48E-04	0.109 (0.051–0.167)	629	2.09E-04	0.110 (0.053–0.169)
hsa-miR-184									
Correlation	822	0.002	-0.108	822	2.86E-05	-0.145	819	1.03E-05	-0.153
Model 1	819	0.074	-0.060 (-0.119–0.005)	819	0.001	-0.099 (-0.157–-0.042)	816	1.76E-04	-0.109 (-0.166–-0.052)
Model 2	728	0.007	-0.088 (-0.151–-0.024)	728	0.004	-0.080 (-0.135–-0.059)	728	0.003	-0.084 (-0.139–-0.028)
hsa-miR-339-3p									
Correlation	850	0.096	-0.057	850	4.44E-05	-0.140	847	4.66E-05	-0.139
Model 1	847	NS	NS	847	0.001	-0.094 (-0.150–-0.039)	844	0.001	-0.095 (-0.150–-0.040)
Model 2	757	0.127	-0.049 (-0.113–0.014)	757	0.006	-0.077 (-0.132–-0.022)	757	0.003	-0.083 (-0.138–-0.028)
hsa-miR-15b-3p									
Correlation	753	0.088	-0.062	753	0.006	-0.099	750	0.008	-0.096
Model 1	751	0.141	-0.050 (-0.116–0.016)	751	0.003	-0.089 (-0.147–-0.030)	748	0.007	-0.082 (-0.140–-0.023)
Model 2	673	NS	NS	673	0.078	-0.053 (-0.111–0.006)	673	0.086	-0.052 (-0.110–0.007)
hsa-miR-93-3p									
Correlation	868	0.656	-0.015	868	0.053	-0.066	865	0.045	-0.068
Model 1	865	NS	NS	865	0.036	-0.060 (-0.115–-0.004)	862	0.019	-0.066 (-0.121–-0.011)
Model 2	773	NS	NS	773	NS	NS	773	NS	NS
hsa-miR-146b-5p									
Correlation	868	0.674	0.014	868	0.011	0.086	865	0.014	0.084
Model 1	865	NS	NS	865	NS	NS	862	NS	NS
Model 2	772	0.144	-0.047 (-0.111–0.016)	772	NS	NS	772	NS	NS
hsa-miR-221-3p									
Correlation	868	0.975	-0.001	868	0.013	0.085	865	0.015	0.082
Model 1	865	NS	NS	865	NS	NS	862	NS	NS
Model 2	772	NS	NS	772	NS	NS	772	NS	NS
hsa-miR-18a-3p									
Correlation	864	0.426	0.027	864	0.277	-0.037	861	0.274	-0.037
Model 1	861	NS	NS	861	NS	NS	858	NS	NS

Model 2	770	NS	NS	770	NS	NS	770	NS	NS
hsa-miR-181a-2-3p									
Correlation	867	0.318	0.034	867	0.129	0.052	864	0.098	0.056
Model 1	864	NS	NS	864	NS	NS	861	NS	NS
Model 2	773	NS	NA	773	NS	NS	773	NS	NS
hsa-miR-642a-5p									
Correlation	868	0.364	0.031	868	0.316	-0.034	865	0.307	-0.035
Model 1	865	NS	NS	865	NS	NS	862	NS	NS
Model 2	772	NS	NS	772	NS	NS	772	NS	NS

***Statistical model:** Correlation = Spearman's correlation; Model 1 = Stepwise regression model including a miRNA of interest (one by one), age, sex, and BMI; Model 2 = Model 1 + leukocyte, erythrocyte, an thrombocyte counts, total cholesterol, LDL, HDL, and triglyceride levels, as well as glycemic status, alcohol consumption, and history of smoking or hypertension. **Note:** The number of samples in the regression models varies according to the number of samples in which the miRNA was expressed and also according to the availability of the variables in the regression models.

Supplementary Table S3. Associations between miRNAs and glycated hemoglobin (HbA1c) levels and percentages. Associations were evaluated with Spearman's correlation and stepwise linear regression models*.

	HbA1c levels			HbA1c %		
	**n**	**p**	**r/β (95% CI)**	**n**	**p**	**r/β (95% CI)**
hsa-miR-144-5p						
Correlation	862	8.05E-05	-0.134	862	5.80E-05	-0.137
Model 1	859	0.009	-0.086 (-0.149–-0.021)	859	0.009	-0.084 (-0.148–-0.021)
Model 2	769	0.051	-0.068 (-0.137–0.000)	769	0.053	-0.068 (-0.136–0.001)
hsa-miR-148a-3p						
Correlation	754	1.39E-05	-0.158	754	9.39E-06	-0.161
Model 1	753	7.72E-05	-0.135 (-0.202–-0.068)	853	6.00E-05	-0.137 (-0.203–-0.070)
Model 2	674	5.05E-05	-0.140 (-0.207–-0.073)	674	3.74E-05	-0.143 (-0.210–-0.075)
hsa-miR-122-5p						
Correlation	702	3.58E-04	0.134	702	2.23E-04	0.139
Model 1	700	0.004	0.105 (0.034–0.177)	700	0.004	0.107 (0.035–0.179)
Model 2	629	0.005	0.104 (0.032–0.176)	629	0.005	0.104 (0.032–0.176)
hsa-miR-184						
Correlation	821	0.434	-0.027	821	0.529	-0.022
Model 1	818	NS	NS	818	NS	NS
Model 2	728	NS	NS	728	NS	NS
hsa-miR-339-3p						
Correlation	849	0.252	-0.039	849	0.330	-0.033
Model 1	846	NS	NS	846	NS	NS
Model 2	757	NS	NS	757	NS	NS
hsa-miR-15b-3p						
Correlation	753	0.001	-0.121	753	1.27E-04	-0.139
Model 1	751	1.80E-04	-0.128 (-0.195–-0.062)	751	5.55E-05	-0.138 (-0.205–-0.071)
Model 2	673	0.065	-0.065 (-0.134–0.004)	673	0.031	-0.075 (-0.144–-0.007)
hsa-miR-93-3p						
Correlation	867	3.29E-06	-0.157	867	5.78E-06	-0.153
Model 1	864	8.73E-07	-0.157 (-0.219–-0.095)	864	1.11E-06	-0.155 (-0.217–-0.093)
Model 2	773	8.21E-06	-0.145 (-0.208–-0.082)	773	1.06E-05	-0.143 (-0.206–-0.080)
hsa-miR-146b-5p						
Correlation	867	3.32E-06	0.157	867	2.39E-06	0.159
Model 1	864	9.57E-05	0.126 (0.063–0.188)	864	9.39E-05	0.126 (0.063–0.188)
Model 2	772	8.05E-05	0.123 (0.058–0.188)	772	0.000258326	0.122 (0.057–0.187)
hsa-miR-221-3p						
Correlation	867	9.62E-05	0.132	867	2.74E-04	0.123
Model 1	864	1.82E-04	0.120 (0.058–0.183)	864	0.001	0.109 (0.046–0.171)
Model 2	772	0.006	0.095 (0.027–0.162)	772	0.014	0.085 (0.018–0.152)
hsa-miR-18a-3p						
Correlation	863	1.30E-04	-0.130	863	1.18E-04	-0.131
Model 1	860	0.001	-0.109 (-0.172–-0.046)	860	0.001	-0.106 (-0.169–-0.042)
Model 2	770	0.002	-0.104 (-0.169–-0.040)	770	0.002	-0.102 (-0.167–-0.037)
hsa-miR-181a-2-3p						
Correlation	866	2.50E-05	0.143	866	2.92E-05	0.142
Model 1	863	1.62E-04	0.121 (0.042–0.184)	863	2.10E-04	0.119 (0.053–0.182)
Model 2	773	0.001	0.110 (0.045–0.174)	773	0.001	0.106 (0.041–0.170)
hsa-miR-642a-5p						
Correlation	867	4.51E-07	0.170	867	7.95E-07	0.167
Model 1	864	8.46E-08	0.171 (0.109–0.233)	871	1.73E-07	0.167 (0.105–0.229)
Model 2	772	8.70E-08	0.176 (0.112–0.239)	772	1.54E-07	0.173 (0.109–0.236)

***Statistical model:** Correlation = Spearman correlation; Model 1 = Stepwise regression model including the miRNA of interest (one by one), age, sex, and BMI; Model 2 = Model 1 + leukocyte, erythrocyte, and thrombocyte counts, total cholesterol, LDL, HDL, and triglyceride levels, as well as glycemic status, alcohol consumption, and history of smoking or hypertension. **Note:** The number of samples in the regression models varies according to the number of samples in which the miRNA was expressed and also according to the availability of the variables in the regression models.

Supplementary Table S4. Associations of miRNAs with serum glucose and insulin levels and the HOMA2 insulin resistance index in normoglycemic (NG) individuals and individuals with impaired fasting glucose (IFG) separately. Associations were evaluated with Spearman's correlation and stepwise linear regression models*.

	**Serum glucose**			**Insulin**			**HOMA2-IR**		
	**n**	**p**	**r/β (95% CI)**	**n**	**p**	**r/β (95% CI)**	**n**	**p**	**r/β (95% CI)**
**In NG individuals**									
hsa-miR-589-3p									
Correlation	532	0.321	-0.043	531	0.007	-0.117	531	0.007	-0.117
Model 1	529	NS	NS	529	0.045	-0.077 (-0.152–-0.002)	529	0.049	-0.075 (-0.150—0.000)
Model 2	511	NS	NS	511	0.028	-0.083 (-0.158–-0.009)	511	0.028	-0.084 (-0.158- -0.009)
hsa-miR-221-3p									
Correlation	531	.656	0.019	531	0.089	0.078	531	0.089	0.074
Model 1	526	NS	NS	529	NS	NS	529	NS	NS
Model 2	511	NS	NS	511	NS	NS	511	NS	NS
hsa-miR-454-5p									
Correlation	441	0.149	0.067	441	0.836	0.010	441	0.818	0.011
Model 1	440	NS	NS	440	NS	NS	440	NS	NS
Model 2	425	NS	NS	425	NS	NS	425	NS	NS
hsa-miR-642a-5p									
Correlation	533	0.651	0.020	533	0.245	-0.050	533	0.248	-0.050
Model 1	531	NS	NS	531	NS	NS	531	NS	NS
Model 2	513	NS	NS	513	NS	NS	513	NS	NS
**In individuals with IFG**									
hsa-miR-885-5p									
Correlation	250	9.00E-05	0.244	250	0.038	0.131	250	0.029	0.138
Model 1	249	0.007	0.169 (0.048–0.291)	249	0.010	0.135 (0.034–0.236)	249	0.007	0.142 (0.040–0.243)
Model 2	237	0.014	0.152 (0.031–0.273)	237	0.016	0.121 (0.024–0.219)	237	0.012	0.127 (0.029–0.226)
hsa-miR-106b-5p									
Correlation	252	9.20E-05	0.244	252	0.048	0.124	252	0.036	0.132
Model 1	251	2.82E-04	0.223 (0.104–0.341)	251	0.090	0.089 (-0.014–0.192)	251	0.064	0.098 (-0.005–0.202)
Model 2	238	0.001	0.197 (0.078–0.316)	238	0.052	0.097 (-0.000–0.195)	238	0.036	0.106 (0.007–0.204)
hsa-miR-122-5p									
Correlation	207	0.407	0.058	207	8.42E-07	0.334	207	9.31E-07	0.333
Model 1	206	NS	NS	206	2.81E-04	0.206 (0.097–0.315)	206	3.00E-04	0.206 (0.096–0.315)
Model 2	196	NS	NS	196	8.83E-04	0.18 (0.076–0.286)	196	0.001	0.180 (0.075–0.286)
hsa-miR-146b-5p									
Correlation	250	0.062	-0.118	250	0.295	0.067	250	0.339	0.061
Model 1	249	0.098	-0.104 (-0.226–0.018)	249	NS	NS	249	NS	NS
Model 2	236	0.018	-0.153 (-0.279—0.027)	236	NS	NS	236	NS	NS

***Statistical model:** Correlation = Spearman's correlation; Model 1 = Stepwise regression model including the miRNA of interest (one by one), age, sex, and BMI; Model 2 = Model 1 + leukocyte, erythrocyte, and thrombocyte counts, total cholesterol, LDL, HDL, and triglyceride levels, as well as glycemic status, alcohol consumption, and history of smoking or hypertension **Note:** The number of samples in the regression models varies according to the number of samples in which the miRNA was expressed and also according to the availability of the variables in the regression models.

Supplementary Table S5. Associations between miRNAs and glycated hemoglobin (HbA1c) levels and percentage in normoglycemic (NG) individuals and individuals with impaired fasting glucose (IFG) separately. Associations were evaluated with Spearman's correlation and stepwise linear regression models*.

	HbA1c			HbA1c %		
	**n**	**p**	**r/β (95% CI)**	**n**	**p**	**r/β (95% CI)**
**In NG individuals**						
hsa-miR-589-3p						
Correlation	531	1.80E-05	-0.185	531	4.80E-05	-0.175
Model 1	529	4.61E-05	-0.167 (-0.246–-0.087)	529	1.37E-04	-0.154 (-0.233–-0.075)
Model 2	511	4.32E-04	-0.146 (-0.227–-0.065)	511	0.001	-0.134 (-0.214–-0.054)
hsa-miR-221-3p						
Correlation	531	4.50E-05	0.176	531	1.29E-04	0.165
Model 1	529	4.05E-06	0.189 (0.109–0.268)	529	7.57E-06	0.181 (0.103–0.260)
Model 2	511	7.31E-05	0.170 (0.087–0.253)	511	9.84E-05	0.165 (0.083–0.247)
hsa-miR-454-5p						
Correlation	441	2.42E-04	-0.174	441	1.34E-04	-0.181
Model 1	440	1.54E-04	-0.170 (-0.257–-0.083)	440	9.94E-05	-0.171 (-0.257–-0.086)
Model 2	425	5.98E-05	-0.180 (-0.267–-0.093)	425	3.9E-05	-0.182 (-0.267–-0.096)
hsa-miR-642a-5p						
Correlation	533	7.80E-05	0.170	533	2.63E-04	0.157
Model 1	531	7.20E-06	0.184 (0.104–0.263)	531	1.9E-05	0.173 (0.094–0.251)
Model 2	513	1.04E-05	0.182 (0.102–0.262)	513	2.42E-05	0.173 (0.093–0.252)
**In individuals with IFG**						
hsa-miR-885-5p						
Correlation	250	0.858	0.011	250	0.812	0.015
Model 1	249	NS	NS	249	NS	NS
Model 2	237	NS	NS	237	NS	NS
hsa-miR-106b-5p						
Correlation	252	0.059	-0.119	252	0.054	-0.121
Model 1	251	0.041	-0.127 (-0.248–-0.006)	251	0.043	-0.125 (-0.246–-0.005)
Model 2	238	0.109	-0.100 (-0.222–0.022)	238	0.095	-0.105 (-0.227–0.024)
hsa-miR-122-5p						
Correlation	207	0.166	0.097	207	0.129	0.106
Model 1	206	NS	NS	206	NS	NS
Model 2	196	NS	NS	196	NS	NS
hsa-miR-146b-5p						
Correlation	250	4.90E-05	0.254	250	6.30E-05	0.250
Model 1	249	2.56E-05	0.260 (0.141–0.378)	249	4.41E-05	0.252 (0.133–0.371)
Model 2	236	5.87E-05	0.259 (0.135–0.384)	236	3.23E-05	0.267 (0.143–0.391)

***Statistical model:** Correlation = Spearman's correlation; Model 1 = Stepwise regression model including the miRNA of interest (one by one), age, sex, and BMI; Model 2 = Model 1 + leukocyte, erythrocyte, and thrombocyte counts, total cholesterol, LDL, HDL, and triglyceride levels, as well as glycemic status, alcohol consumption, and history of smoking or hypertension **Note:** the number of samples in the regression models varies according to the number of samples in which the miRNA was expressed and also according to the availability of the variables in the regression models.

Supplementary Table S6. Significant associations between the miRNAs of interest and their predicted targets in the insulin signaling pathway (KEGG hsa04910). Only predicted targets with a p<0.05 in Spearman's correlation and the linear regression model* are presented.

miRNA/gene ID	Symbol in Figure 2	Probe ID	Spearman's correlation				Linear regression model*			
			n	p	p_c	r	n	p	β	95%CI
hsa-miR-181a-2-3p										
CRK	CRK	7040014	739	0.001	0.075	-0.125	678	0.014	-0.094	-0.169 – -0.020
CBL	CBL	4120037	739	0.002	0.229	-0.113	678	0.023	-0.086	-0.160 – -0.012
let-7a										
MKNK2	MNK	5050553	739	0.001	0.106	-0.121	677	4.60E-04	-0.136	-0.212 – -0.060
HK2	HK	7150661	739	0.001	0.155	0.117	677	0.021	0.085	0.013 – 0.158
PRKAB2	PHK	5860307	739	0.015	1.000	0.089	677	0.012	0.097	0.022 – 0.172
RAPGEF1	CRF2	6270037	739	0.043	1.000	0.074	677	0.036	0.082	0.006 – 0.157
miR-146b-5p										
PRKAG2	PKA	1050196	741	9.67E-07	1.07E-04	-0.179	679	1.07E-06	-0.192	-0.268 – -0.115
PRKAG2	PKA	3120279	741	8.32E-05	0.009	-0.144	679	0.001	-0.133	-0.210 – -0.057
RPS6KB2	RPS6KB	4150136	741	0.001	0.148	0.118	679	0.005	0.109	0.033 – 0.185
MAPK1	ERK1/2	2570026	741	0.001	0.151	-0.117	679	6.30E-05	-0.149	-0.222 – -0.076
MKNK2	MKNK	940152	741	0.002	0.207	-0.114	679	0.001	-0.127	-0.203 – -0.051
IRS2	IRS	6110736	741	0.005	0.573	-0.103	679	0.022	-0.087	-0.161 – -0.013
MAPK1	ERK1/2	3180398	741	0.019	1.000	-0.086	679	0.003	-0.113	-0.188 – -0.038
miR-148a-3p										
HK1	HK	1660196	633	2.72E-04	0.030	0.144	586	4.15E-05	0.163	0.085 – 0.240
MAP2K1	MEK1/2	1050600	633	0.003	0.299	0.119	586	0.030	0.084	0.008 – 0.161
PRKCZ	aPKC	2750324	633	0.033	1.000	-0.085	586	0.040	-0.084	-0.163 – -0.004
PHKG2	PHK	3310358	633	0.036	1.000	-0.083	586	0.046	-0.080	-0.159 – -0.001
miR-221-3p										
PPP1CB	PP1	6200369	741	1.57E-06	1.74E-04	-0.175	679	7.98E-07	-0.187	-0.260 – -0.113
PHKA2	PHK	1050059	741	2.61E-06	2.90E-04	0.172	679	5.76E-06	0.179	0.102 – 0.255
PYGB	PYG	4540326	741	3.32E-05	0.004	0.152	679	5.93E-05	0.152	0.079 – 0.226
RPS6KB1	p70S6K	1940576	741	3.60E-05	0.004	-0.151	679	2.57E-04	-0.142	-0.218 – -0.066
ACACB	ACC	2230678	741	4.35E-05	0.005	0.150	679	1.40E-06	0.192	0.115 – 0.270
CBL	CBL	6960209	741	1.35E-04	0.015	0.140	679	5.10E-05	0.153	0.079 – 0.226
CALM1	PHK	1660477	741	2.82E-04	0.031	0.133	679	2.10E-04	0.142	0.068 – 0.217
PIK3CG	PI3K	3130136	741	3.00E-04	0.033	0.132	679	0.021	0.087	0.013 – 0.160
ACACA	ACC	2630692	741	0.002	0.212	0.114	679	0.001	0.123	0.048 – 0.199
PRKAG2	PKA	3120279	741	0.002	0.248	-0.112	679	0.011	-0.099	-0.175 – -0.022
SOCS4	SOCS	7100082	741	0.002	0.264	-0.111	679	0.024	-0.089	-0.166 – -0.012
ACACA	ACC	2190414	741	0.006	0.648	0.101	679	0.001	0.132	0.054 – 0.211
PIK3R1	PI3K	6520630	741	0.008	0.834	0.098	679	0.005	0.114	0.035 – 0.192
IKBKB	IKK	6660373	741	0.008	0.874	0.098	679	0.008	0.103	0.027 – 0.179
PYGL	PYG	2760427	741	0.011	1.000	0.094	679	0.047	0.072	0.001 – 0.143
PRKAA1	PKA	6980750	741	0.014	1.000	-0.090	679	0.009	-0.103	-0.179 – -0.026
PRKCZ	aPKC	2750324	741	0.015	1.000	0.090	679	0.007	0.107	0.030 – 0.185
CBL	CBL	4120037	741	0.022	1.000	0.084	679	0.016	0.091	0.017 – 0.166
miR-589-3p										
PPP1CB	PP1	6200369	738	0.001	0.136	0.119	676	0.001	0.125	0.050 – 0.200
PRKAA1	PKA	4150079	738	0.003	0.371	0.108	676	0.010	0.099	0.023 – 0.175
PRKAA1	PKA	6980750	738	0.006	0.720	0.100	676	0.001	0.129	0.052 – 0.205
PYGL	PYG	2760427	738	0.011	1.000	-0.093	676	0.049	-0.073	-0.145 – -0.001

***Statistical model**: Stepwise AIC linear regression model including the miRNA of interest, age, sex, BMI, the leukocyte, erythrocyte, and thrombocyte count, glycemic status, as well as glucose, insulin, and HbA1c levels, HbA1c%, and the HOMA2 IR index **Note:** The number of samples in the regression models varies according to the number of samples in which the miRNA was expressed and also according to the availability of the variables in the regression models.

Supplementary Table S7. Significant associations between the miRNAs of interest and their predicted targets in the type 2 diabetes mellitus pathway (KEGG hsa04930). Only predicted targets with a p<0.05 in Spearman's correlation and the linear regression model* are presented.

	**Symbol in Figure 2**		**Spearman's correlation**				**Linear regression model***			
**miRNA/gene ID**		**Accession**	**n**	**p**	**p_c**	**r**	**n**	**p**	**β**	**95%CI**
hsa-miR-181a-2-3p										
PIK3R1	PI3K	1470753	739	0.004	0.124	0.106	678	0.034	0.083	0.006 – 0.160
MAPK3	ERK	2060561	739	0.012	0.392	-0.093	678	0.038	-0.080	-0.156 – -0.004
let-7a										
PKM2	PYK	2030093	739	0.006	0.194	-0.101	677	0.007	-0.105	-0.181 – -0.029
HK2	HK	7150661	739	0.001	0.046	0.117	677	0.021	0.085	0.013 – 0.158
PRKCZ	PKCζ	130551	739	0.028	0.922	0.081	677	0.050	0.078	0.000 – 0.156
miR-146b-5p										
SOCS2	SOCs	6770673	741	1.01E-05	3.34E-04	0.161	679	3.46E-04	0.134	0.061 – 0.207
PKM2	PYK	160170	741	5.50E-05	0.002	0.148	679	2.48E-05	0.159	0.086 – 0.233
MAPK1	ERK	2570026	741	0.001	0.045	-0.117	679	6.30E-05	-0.149	-0.222 – -0.076
IRS2	IRS	6110736	741	0.005	0.170	-0.103	679	0.022	-0.087	-0.161 – -0.013
MAPK1	ERK	3180398	741	0.019	0.615	-0.086	679	0.003	-0.113	-0.188 – -0.038
PKM2	PYK	2030093	741	0.020	0.657	0.086	679	0.018	0.094	0.016 – 0.171
miR-148a-3p										
HK1	HK	1660196	633	2.72E-04	0.009	0.144	586	4.15E-05	0.163	0.085 – 0.240
PRKCZ	PKCζ	2750324	633	0.033	1.000	-0.085	586	0.040	-0.084	-0.163 – -0.004
miR-221-3p										
PKM2	PYK	160170	741	4.02E-06	1.33E-04	0.168	679	1.35E-05	0.167	0.093 – 0.242
MAPK3	ERK	3870601	741	1.62E-04	0.005	0.138	679	5.22E-05	0.156	0.081 – 0.231
PIK3CG	PI3K	3130136	741	3.00E-04	0.010	0.132	679	0.021	0.087	0.013 – 0.160
PKM2	PYK	2030093	741	3.56E-04	0.012	0.131	679	2.75E-05	0.163	0.088 – 0.239
SOCS4	SOCS	7100082	741	0.002	0.078	-0.111	679	0.024	-0.089	-0.166 – -0.012
PIK3R1	PI3K	6520630	741	0.008	0.248	0.098	679	0.005	0.114	0.035 – 0.192
IKBKB	IKK	6660373	741	0.008	0.260	0.098	679	0.008	0.103	0.027 – 0.179
PRKCZ	PKCζ	2750324	741	0.015	0.485	0.090	679	0.007	0.107	0.030 – 0.185
PRKCD	PKCε/δ	6580039	741	0.031	1.000	0.079	679	0.003	0.115	0.039 – 0.192

***Statistical model**: Stepwise AIC linear regression model including the miRNA of interest, age, sex, BMI, the leukocyte, erythrocyte, and thrombocyte count, glycemic status, glucose, insulin, and HbA1c levels, as well as HbA1c% and the HOMA2 IR index. **Note:** The number of samples in the regression models varies according to the number of samples in which the miRNA and mRNA were expressed.

Supplementary Table S8. Associations of serum hsa-miR-122-5p (form the subpopulation of the YFS) with serum glucose and insulin levels, as well as the HOMA2 insulin resistance (IR) index, HbA1c, and HbA1c%. Associations were evaluated with Spearman's rank correlation.

	**n**	**p-value**	**r**
Glucose	137	$2.36*10^{-5}$	0.353
Insulin	137	$5.54*10^{-7}$	0.412
HOMA2-IR	137	$3.56*10^{-7}$	0.419
HbA1c	137	0.851	0.016
HbA1c %	137	0.758	0.027

Supplementary Table S9.

Previously reported associations between the miRNAs of interest and phenotypes related to type 2 diabetes

	Circulatory levels affected by				Tissue levels affected by*	Associated with		Regulates		Is regulated by
	T2D	T1D	Pre-diabetes	Fatty liver	T2D	Obesity/ Adipocytes	Inflammation	Insulin secretion	Glucose metabolism	Glucose levels
let-7a-5p	↓[1]									
miR-15b-3p	↓[2]				Yes[3] (Muscle)			Yes[4]		
miR-18a-3p	↓[5]									
miR-93-3p		↓[6]	↓[7,8]			Yes[9]			Yes[8]	Yes[10]
miR-122-5p				↑[11]						
miR-144-5p	↑[12-14]								Yes[14-15]	
miR-146b-5p						Yes[16]	Yes[17]			
miR-148a-3p	↑[18]	↑[19]	↓[18]			Yes[20]				
miR-181a-2-3p	↑[21]	↑[19]								
miR-184								Yes[22,23]		Yes[24]
miR-221-3p					Yes (Artery wall)[25]		Yes[17]			Yes[26,27]
miR-339-3p									Yes[28]	
miR-885-5p				↑[11]						
miR-589-3p										
miR-642a-5p						Yes[29]				

* Tissue in parenthesis

[1-8], o[9, 10], o[11]0[12-14] 0[14, 15] 0[16, 17] o[18, 19], o[20] , [21],o [22, 23], p[24], p[25], ö[17], o[26, 27], o[28], [29]

Supplementary Materials and Methods

The Young Finns Study (YFS)

The YFS is a multicenter follow-up study on cardiovascular risk from childhood to adulthood in Finland. The YFS was launched in 1980, when 3,596 children and adolescents (aged 3–18 years) participated in the baseline study [30]. The subjects were randomly selected from the Finnish national register among the chosen age groups and the five study districts. Thereafter, the subjects have been followed (in 1986, 2001, 2007, and 2011) with several examinations, including comprehensive risk factor assessments. The 30-year follow-up was performed in 2011, with 2,063 adults, aged 34–49 years, participating in the study. The examinations included physical measurements, blood tests, and questionnaires. Participants in the follow-up studies have been found to be more often women and older than those who dropped out, but no significant differences in risk factors have been found[30]. The present study has been approved by the 1st Ethical Committee of the Hospital District of Southwest Finland on September 21st, 2010, and by local ethical committees (1st Ethical Committee of the Hospital District of Southwest Finland, Regional Ethics Committee of the Expert Responsibility area of Tampere University Hospital, the Helsinki University Hospital Ethical Committee of Medicine, the Research Ethics Committee of the Northern Savo Hospital District. and the Ethics Committee of the Northern Ostrobothnia Hospital District). All study subjects gave an informed consent, and the study was conducted according to the principles of the Declaration of Helsinki. In the earlier follow-ups involving also underage participants, informed consent was obtained from a parent and/or legal guardian. The YFS samples for miRNA analysis (n=992) were selected independently of glycemic status from individuals with the most comprehensive data on risk factors, metabonomics, transcriptomics, genome-wide genotyping, and other phenotyping in the follow-up studies performed in 1986, 2001, 2007, and 2011. After quality control, the study population comprises 871 individuals with successful miRNA profiling (demographics in Supplementary Table 1).

Clinical and biochemical measurements

Weight and height were measured, and BMI was calculated as weight(kg)/height(m)^2. Waist circumference was measured to the nearest 0.1 cm. Blood pressure was measured with a random zero sphygmomanometer. Venous blood samples were drawn from the right antecubital vein after an overnight fast. For blood count analysis, whole blood was anticoagulated with EDTA. Blood cell parameters were measured with flow cytometric particle counting (cells) and photometry (Hb) using Sysmex XE- 5000 and XT-2000i analyzers (Sysmex Corporation) with reagents provided by the manufacturer (Cellpack and Sulfolyser).

For the biochemical measurements, serum was separated, aliquoted, and stored at -70°C until analysis. Glucose, cholesterol, and triglyceride concentrations were measured with Glucose, Cholesterol, and Triglycerides System Reagent (Beckman Coulter Biomedical). The serum triglyceride concentration was assayed using the enzymatic glycerol kinase–glycerol phosphate oxidase method (Beckman Coulter Biomedical). Serum total cholesterol levels were measured by the enzymatic cholesterol esterase–cholesterol oxidase method (Beckman Coulter Biomedical). The same reagent was used for estimating HDL cholesterol levels after the precipitation of low-density lipoprotein (LDL) and very low-density lipoprotein (VLDL) with dextran sulfate- Mg^{2+}. Serum glucose concentrations were determined by the enzymatic hexokinase method (Beckman Coulter Biomedical). All the above-mentioned assays were performed on an AU400 instrument (AU400, Olympus).

Glycated hemoglobin (HbA1c) fraction in whole blood was measured by an Abbott Architect ci8200 analyzer (Abbott Laboratories). The concentration of total hemoglobin was first determined colorimetrically, after which the concentration of HbA1c was measured immunoturbidimetrically using the microparticle agglutination inhibition method (Fisher Diagnostics). These two concentrations were used to calculate the HbA1c percentage (HbA1c%). Insulin levels were measured with a microparticle enzyme immunoassay kit (Abbott Laboratories,

Chicago, IL), and the HOMA2 index was calculated according to the online HOMA2-IR calculator (https://www.dtu.ox.ac.uk/homacalculator/).

Categorizing individuals according to T2D and glycemic status

Individuals were categorized into the normoglycemic (NG), IFG, and T2D groups. The classification of IFG was based on fasting serum glucose and HbA1c according to the criteria of the WHO[31]. Individuals with IFG, were defined as having a fasting serum glucose level of 5.60–6.99 mmol/l or a HbA1c of 5.7%–6.4% (38–46 mmol/mol) and not diagnosed with T2D. The diagnosis of T2D included subjects with a fasting plasma glucose level of over 7.0 mmol/l or HbA1c of over 6.5% (48 mmol/mol), or those with reported use of oral glucose-lowering medication or insulin (but had not reported having type 1 diabetes) or who had a reported diagnosis of T2D by a physician. Individuals with type 1 diabetes were discarded from analysis.

Whole blood RNA isolation, quality control, and miRNA expression profiling

Whole blood (2.5 ml) was collected into PaXgene Blood RNA Tubes (PreAnalytix). The tubes were inverted 8–10 times and then stored at room temperature for at least 2 hours. The tubes were frozen (-80°C) and thawed overnight before RNA isolation (both miRNA and total RNA) with a PAXgene Blood MicroRNA Kit (Qiagen) including the DNase Set using the QiaCube extraction robot. The concentrations and purity of the RNA samples were evaluated spectrophotometrically (BioPhotomer, Eppendorf). The RNA isolation process was validated by analyzing the integrity of several RNAs with the RNA 6000 Nano Chip Kit (Agilent). The presence of the small RNA fraction was confirmed by the Agilent Small RNA Kit (Agilent).

MicroRNA expression profiling was performed with the TaqMan® OpenArray® MicroRNA Panel (Applied Biosystems) containing 758 microRNAs. In brief, 100 ng of RNA was used to run both A and B pools of Megaplex (Applied Biosystems) preamplification for cDNA synthesis. In the OpenArray Sample Loading Plate, 22.5 µl of each preamplified pool was mixed 1:1 with TaqMan OpenArray Real-Time PCR Master Mix. MicroRNA panels were loaded using the AccuFill System and run with the QuantStudio 12K Flex (Applied Biosystems).

Primary data analysis was performed with Expression Suite Software version 1.0.1. As recommended by the manufactures of the miRNA panels, RNU6, RNU44, and RNU48 were used as housekeeping small RNAs. Assays with an Amplification score of >1 and Cq Confidence of >0.7 were accepted. Ninety-five samples were excluded due to a low number of miRNAs expressed (≤200 miRNAs per sample), and in further analysis, 243 miRNAs that were expressed in at least 2/3 of the samples were included. The number of miRNAs present in detectable levels in the majority of the blood samples was well in line with a previous similar analysis of blood tissue [32]. The RNA quality and functionality of the TaqMan OpenArray microRNA expression panels have been validated previously [33]. After quality control and removal of outlier miRNAs, profiling was successful on 871 samples. To correct for batch effects, the principal component analysis was performed for the miRNA expression data. The data was adjusted for 10 of the first 20 principal components from the principal component analysis.

Serum RNA isolation and miRNA expression profiling

For serum miRNA isolation, the serum was thawed, and miRNAs were isolated from 200 µl of serum with the miRNeasy Serum/Plasma Kit (Qiagen) including the DNase Set using the QiaCube. MicroRNA expression profiling was performed with the same TaqMan® OpenArray® MicroRNA Panel (Life Technologies) as from whole blood, but scaled-down Low Sample Protocol was used for serum samples. In comparison to the standard protocol, 10 µl of the miRNA eluate was used in cDNA synthesis for both A and B pools of Megaplex, and 7.5 µl of cDNA was the preamplified (total reaction volume 18 µl) and diluted with the final dilution ratio of 1:10 with nuclease free water and TE buffer.

Also, for serum data, only assays with an Amplification score of >1 and Cq Confidence of >0.7 were accepted, and all 146 samples passed quality control. Due to the lower number of samples, miRNAs expressed in at least ½

of the samples were included in the data. The serum data was normalized with the global mean normalization approach in R language4. The approach involves the calculation of a normalization factor as the global mean of all expressed miRNAs per sample. Further, between-sample normalization was performed with the quantile normalization method. Batch correction, when needed, was accomplished with ComBat5.

Genome-wide expression analysis (transcriptomics)

The expression levels were analyzed with an Illumina HumanHT-12 version 4 Expression BeadChip (Illumina Inc.). Utilizing the same RNA sample for both mRNA and miRNA expression profiling, 200 ng of RNA was reverse-transcribed into cDNA and biotin-UTP-labeled using the Illumina TotalPrep RNA Amplification Kit (Ambion); 1,500 ng of cDNA was then hybridized onto the Illumina HumanHT-12 v4 Expression BeadChip. The BeadChips were scanned with the Illumina iScan system. Raw Illumina probe data was exported from Genomestudio and analyzed in R (http://www.r-project.org/) using the Bioconductor (http://www.bioconductor.org/) packages. The expression data was processed using nonparametric background correction, followed by quantile normalization with control and expression probes, using the neqc function in the limma package and log2 transformation. The data processing has been described in more detailed in an article by Elovainio et al. [34]. The expression analysis was successful in 743 of the 871 samples with a miRNA expression profile.

Statistical analysis

MicroRNA expressions differences over glycemic status groups were analyzed by Kruskal-Wallis analysis of variance, after which the different glycemic status groups (i.e., NG vs. IFG, NG vs. T2D and IFG vs. T2D) were compared by using the Mann-Whitney U test. In order to take account of the multiple testing, conservative Bonferroni-corrected p-values (p_c-value) were calculated, and a p_c-value of <0.05 ($=p<0.00021$) was considered significant. For dysregulated miRNAs, fold changes (FCs) were calculated for each individual sample in comparison to the average of all NG individuals. To analyze whether dysregulated miRNAs were independent predictors of IFG or T2D, a stepwise Akaike information criterion (AIC) logistic regression model was utilized. Two different models were used as follows: <u>Model 1 = the</u> dependent variable was glycemic status (IFG/T2D vs. NG and T2D vs. IFG, one by one) predicted with the discovered miRNAs (forced in to the model one by one), age, sex, and BMI; and <u>Model 2</u> = Model 1 + the leukocyte, erythrocyte, and thrombocyte count, as well as total cholesterol, LDL, HDL and triglyceride levels, in addition to alcohol consumption and the history of smoking and hypertension. In regression models, $p<0.05$ was considered significant. The number of samples in the regression models varied according to the number of samples in which the miRNA was expressed and also according to the availability of the variables in the regression models, as all measurements were not successful/available from all of the samples.

The association of miRNA levels with glucose, insulin, HbA1c (mmol/mol), HbA1c%, and the HOMA2 IR index were correlated with Spearman's rank-order correlation. P_c-value<0.05 was considered significant. Independent prediction value was evaluated with linear regression models. <u>Model 1</u>: dependent variable one by one (i.e. glucose, HbA1c, HbA1c%, insulin, and HOMA2 IR index) predicted with the discovered miRNAs (forced in to the model one by one), as well as age, sex, and BMI; and Model 2: Model 1 + the leukocyte, erythrocyte, and thrombocyte count, as well as total cholesterol, LDL, HDL, and triglyceride levels, in addition to alcohol consumption, the history of smoking and hypertension, and glycemic status (NG/IFG/T2D). Glycemic status–stratified analyzes were performed as in the whole population, with the exception of glycemic status not being included in Model 2. In regression models, $p<0.05$ was considered significant.

To analyze the co-regulation of the miRNAs of interest and the existence of possible expression clusters of these miRNAs, between-miRNA Spearman's rank-order correlations were analyzed. Also, to validate our results, we analyzed the differences in hsa-miR-144-5p and hsa-let-7a-5p over the glycemic status groups from the miRNA data profiled with Human MiRNA Microarray Release 14.0, 8 × 15 K (Agilent) from whole blood of 72 individuals included in the YFS 2011 follow-up. The sample preparation, miRNA profiling, and data

preprocessing have been described previously[20]. Differences in microRNA expressions between the glycemic status groups were analyzed as with the larger data set from whole blood, and hsa-miR-144-5p and hsa-let-7a-5p were selected as they were available in both arrays and gave significant results in the non-parametric tests.

To analyze the possibility that miRNAs associated with the individuals' glycemic status, glucose levels, or indicators of insulin resistance are expressed particularly in certain circulatory blood cells, we correlated the levels of these miRNAs with the leukocyte, erythrocyte, and thrombocyte counts using Spearman's rank-order correlation. In addition, we investigated whether some of the miRNAs were originating solely from serum by replicating the non-parametric tests and the correlations between the miRNAs of interest and glycemic status and the indicators of glucose metabolism in the serum miRNA profilings performed with TaqMan® OpenArray® MicroRNA Panels from a subpopulation (n=146) of the YFS (see supplementary materials and methods).

In the target mRNA analysis, Spearman's rank-order correlations were performed between the FCs of the miRNAs of interest (miRNAs with significantly different levels between glycemic status groups or significant correlation with glucose, insulin, HbA1c, HbA1c% levels, or the HOMA2 index in whole population) and the expression of their predicted targets (predicted by two or more algorithms according to miRGator v.3.0, http://mirgator.kobic.re.kr/). Transcripts with a correlation $p<0.05$ were included in the pathway enrichment analysis performed for the KEGG pathways in the molecular signatures database (http://software.broadinstitute.org/gsea/msigdb/index.jsp). Pathways with an FDR-q value of <0.05 were considered significantly enriched with target mRNAs. The most significant pathways were further analyzed for coregulation of all the miRNAs of interest. All predicted targets (predicted by at least one algorithm in miRGator 3.0) from at least one of the miRNAs of interest were selected from the most significantly enriched pathways and correlated (Spearman's rank order correlation) with their predicted regulatory miRNA levels. The predicted target mRNAs with significant correlation ($p<0.05$) were included in further analysis. The independent statistical prediction values of the miRNAs of interest on the target mRNA levels were evaluated with an AIC linear regression model including the miRNA of interest, age, sex, BMI, the leukocyte, erythrocyte, and thrombocyte count, glycemic status, as well as glucose, insulin, and HbA1c levels, and HbA1c% and the HOMA2 IR index. Transcripts whose levels were significantly ($p<0.05$) and independently statistically predicted by the levels of the miRNAs of interest were considered to be affected by these miRNAs.

Availability of the data

The datasets generated and/or analyzed during the current study are not publicly available due to restrictions imposed by Finnish legislation. However, data sharing is possible upon reasonable request and all decisions are made by the YFS Publication and Data Sharing board.

Supplementary references

References

1. Santovito, D. *et al*. Plasma exosome microRNA profiling unravels a new potential modulator of adiponectin pathway in diabetes: effect of glycemic control. *J. Clin. Endocrinol. Metab.* **99**, E1681-5 (2014).

2. Pescador, N. *et al*. Serum circulating microRNA profiling for identification of potential type 2 diabetes and obesity biomarkers. *PLoS One* **8**, e77251 (2013).

3. Bork-Jensen, J. *et al*. Glucose tolerance is associated with differential expression of microRNAs in skeletal muscle: results from studies of twins with and without type 2 diabetes. *Diabetologia* **58**, 363-373 (2015).

4. Su, Y. *et al*. Maternal Low Protein Isocaloric Diet Suppresses Pancreatic beta-Cell Proliferation in Mouse Offspring via miR-15b. *Endocrinology* **157**, 4782-4793 (2016).

5. Mao, G. & Liu, L. microRNA-18a is a genetic marker for the early diagnosis of cerebral injury induced by type 2 diabetes. *Exp. Ther. Med.* **8**, 1901-1905 (2014).

6. Nunez Lopez, Y. O., Garufi, G. & Seyhan, A. A. Altered levels of circulating cytokines and microRNAs in lean and obese individuals with prediabetes and type 2 diabetes. *Mol. Biosyst* **13**, 106-121 (2016).

7. Salas-Perez, F. *et al*. MicroRNAs miR-21a and miR-93 are down regulated in peripheral blood mononuclear cells (PBMCs) from patients with type 1 diabetes. *Immunobiology* **218**, 733-737 (2013).

8. Yan, S. T. *et al*. MiR-199a is overexpressed in plasma of type 2 diabetes patients which contributes to type 2 diabetes by targeting GLUT4. *Mol. Cell. Biochem.* **397**, 45-51 (2014).

9. Wu, Y. *et al*. Identification of miR-106b-93 as a negative regulator of brown adipocyte differentiation. *Biochem. Biophys. Res. Commun.* **438**, 575-580 (2013).

10. Long, J., Wang, Y., Wang, W., Chang, B. H. & Danesh, F. R. Identification of microRNA-93 as a novel regulator of vascular endothelial growth factor in hyperglycemic conditions. *J. Biol. Chem.* **285**, 23457-23465 (2010).

11. Raitoharju, E. *et al*. Blood hsa-miR-122-5p and hsa-miR-885-5p levels associate with fatty liver and related lipoprotein metabolism-The Young Finns Study. *Sci. Rep.* **6**, 38262 (2016).

12. Wang, X. *et al*. Determination of 14 circulating microRNAs in Swedes and Iraqis with and without diabetes mellitus type 2. *PLoS One* **9**, e86792 (2014).

13. Yan, S. *et al*. Differential expression of microRNAs in plasma of patients with prediabetes and newly diagnosed type 2 diabetes. *Acta Diabetol.* **53**, 693-702 (2016).

14. Karolina, D. S. *et al*. MicroRNA 144 impairs insulin signaling by inhibiting the expression of insulin receptor substrate 1 in type 2 diabetes mellitus. *PLoS One* **6**, e22839 (2011).

15. Liu, M., Gao, J., Huang, Q., Jin, Y. & Wei, Z. Downregulating microRNA-144 mediates a metabolic shift in lung cancer cells by regulating GLUT1 expression. *Oncol. Lett.* **11**, 3772-3776 (2016).

16. Ahn, J., Lee, H., Jung, C. H., Jeon, T. I. & Ha, T. Y. MicroRNA-146b promotes adipogenesis by suppressing the SIRT1-FOXO1 cascade. *EMBO Mol. Med.* **5**, 1602-1612 (2013).

17. Marques-Rocha, J. L. *et al.* Noncoding RNAs, cytokines, and inflammation-related diseases. *FASEB J.* **29**, 3595-3611 (2015).

18. Seyhan, A. A. *et al.* Pancreas-enriched miRNAs are altered in the circulation of subjects with diabetes: a pilot cross-sectional study. *Sci. Rep.* **6**, 31479 (2016).

19. Nielsen, L. B. *et al.* Circulating levels of microRNA from children with newly diagnosed type 1 diabetes and healthy controls: evidence that miR-25 associates to residual beta-cell function and glycaemic control during disease progression. *Exp. Diabetes Res.* **2012**, 896362 (2012).

20. Shi, C. *et al.* Adipogenic miRNA and meta-signature miRNAs involved in human adipocyte differentiation and obesity. *Oncotarget* **7**, 40830-40845 (2016).

21. Zhou, B. *et al.* Downregulation of miR-181a upregulates sirtuin-1 (SIRT1) and improves hepatic insulin sensitivity. *Diabetologia* **55**, 2032-2043 (2012).

22. Bolmeson, C. *et al.* Differences in islet-enriched miRNAs in healthy and glucose intolerant human subjects. *Biochem. Biophys. Res. Commun.* **404**, 16-22 (2011).

23. Morita, S., Horii, T., Kimura, M. & Hatada, I. MiR-184 regulates insulin secretion through repression of Slc25a22. *PeerJ* **1**, e162 (2013).

24. Tattikota, S. G. *et al.* miR-184 Regulates Pancreatic beta-Cell Function According to Glucose Metabolism. *J. Biol. Chem.* **290**, 20284-20294 (2015).

25. Coleman, C. B. *et al.* Elevation of miR-221 and -222 in the internal mammary arteries of diabetic subjects and normalization with metformin. *Mol. Cell. Endocrinol.* **374**, 125-129 (2013).

26. Li, Y. *et al.* MicroRNA-221 regulates high glucose-induced endothelial dysfunction. *Biochem. Biophys. Res. Commun.* **381**, 81-83 (2009).

27. Silambarasan, M. *et al.* MicroRNAs in Hyperglycemia Induced Endothelial Cell Dysfunction. *Int. J. Mol. Sci.* **17**, 518 (2016).

28. Jia, Y. *et al.* Maternal low-protein diet induces gender-dependent changes in epigenetic regulation of the glucose-6-phosphatase gene in newborn piglet liver. *J. Nutr.* **142**, 1659-1665 (2012).

29. Zaragosi, L. E. *et al.* Small RNA sequencing reveals miR-642a-3p as a novel adipocyte-specific microRNA and miR-30 as a key regulator of human adipogenesis. *Genome Biol.* **12**, R64-2011-12-7-r64 (2011).

30. Raitakari, O. T. *et al.* Cohort profile: the cardiovascular risk in Young Finns Study. *International journal of epidemiology* **37**, 1220-6 (2008).

31. World Health Organization. Global report on diabetes. *Geneva* (2016).

32. Leidinger, P., Backes, C., Meder, B., Meese, E. & Keller, A. The human miRNA repertoire of different blood compounds. *BMC Genomics* **15**, 474-2164-15-474 (2014).

33. Raitoharju, E. *et al*. Blood microRNA profile associates with the levels of serum lipids and metabolites associated with glucose metabolism and insulin resistance and pinpoints pathways underlying metabolic syndrome: The cardiovascular risk in Young Finns Study. *Mol. Cell. Endocrinol.* **391**, 41-49 (2014).

34. Elovainio, M. *et al*. Activated immune-inflammatory pathways are associated with long-standing depressive symptoms: Evidence from gene-set enrichment analyses in the Young Finns Study. *J. Psychiatr. Res.* **71**, 120-125 (2015).
